# MiR-431 attenuates synaptic plasticity and memory deficits in APPswe/PS1dE9 mice

**DOI:** 10.1172/jci.insight.166270

**Published:** 2023-06-22

**Authors:** Jianwei Ge, Zhiwei Xue, Shu Shu, Linjie Yu, Ruomeng Qin, Wenyuan Tao, Pinyi Liu, Xiaohong Dong, Zhen Lan, Xinyu Bao, Lei Ye, Yun Xu, Xiaolei Zhu

**Affiliations:** 1 Department of Neurology, Nanjing Drum Tower Hospital, Affiliated Hospital of Medical School, Nanjing University, Nanjing, China.; 2 State Key Laboratory of Pharmaceutical Biotechnology and Institute of Translational Medicine for Brain Critical Diseases, Nanjing University, Nanjing, China.; 3 Institute of Brain Sciences, Institute of Brain Disorder Translational Medicine, Nanjing University, Nanjing, China.; 4 Jiangsu Key Laboratory for Molecular Medicine, Medical School of Nanjing University, Nanjing, China.; 5 Nanjing Neuropsychiatry Clinic Medical Center, Nanjing, China.; 6 Department of Neurology, Drum Tower Hospital of Nanjing Medical University, Nanjing, China.

**Keywords:** Aging, Therapeutics, Neurodegeneration

## Abstract

Synaptic plasticity impairment plays a critical role in the pathogenesis of Alzheimer’s disease (AD), and emerging evidence has shown that microRNAs (miRs) are alternative biomarkers and therapeutic targets for synaptic dysfunctions in AD. In this study, we found that the level of miR-431 was downregulated in the plasma of patients with amnestic mild cognitive impairment and AD. In addition, it was decreased in the hippocampus and plasma of APPswe/PS1dE9 (APP/PS1) mice. Lentivirus-mediated miR-431 overexpression in the hippocampus CA1 ameliorated synaptic plasticity and memory deficits of APP/PS1 mice, while it did not affect amyloid-β levels. Smad4 was identified as a target of miR-431, and Smad4 knockdown modulated the expression of synaptic proteins, including SAP102, and protected against synaptic plasticity and memory dysfunctions in APP/PS1 mice. Furthermore, Smad4 overexpression reversed the protective effects of miR-431, indicating that miR-431 attenuated synaptic impairment at least partially by Smad4 inhibition. Thus, these results indicated that miR-431/Smad4 might be a potential therapeutic target for AD treatment.

## Graphical abstract

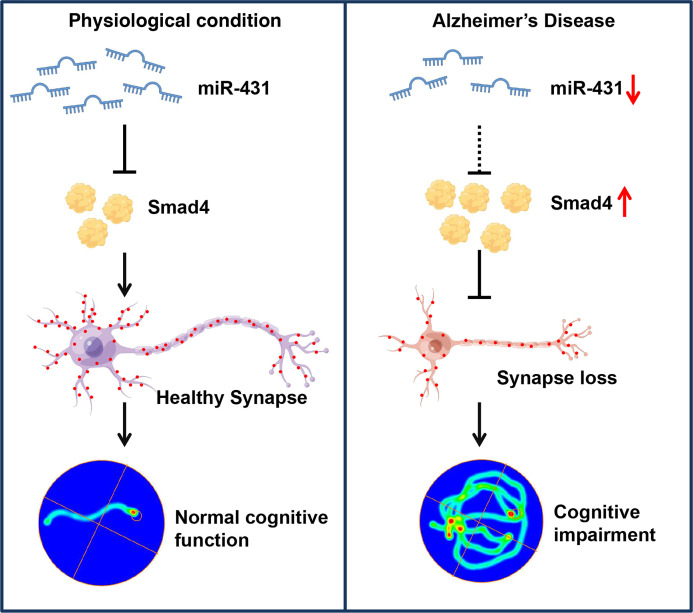

## Introduction

Alzheimer’s disease (AD), an age-dependent neurodegenerative disease, is characterized by memory loss and progressive cognitive function decline. A cross-sectional study in China has shown that the overall prevalence of AD is estimated to be 3.9%, representing 10 million individuals aged 60 years and older with AD in China (1). The annual cost is $19,144.36 per patient with AD in China in 2015, and the total cost is estimated to be $507.49 billion in 2030 (2). Although the pathological mechanisms of AD are not fully understood, synaptic loss is considered an early pathological marker of AD and closely correlates with the cognitive impairment in AD (3). Thus, reversing synaptic dysfunctions might rescue the memory deficits associated with AD.

MicroRNAs (miRNAs), a group of small noncoding RNAs with the length of 18–22 nucleotides, inhibit protein translation through binding with the 3′-untranslated region (3′-UTR) of target mRNAs and play a crucial part in AD pathogenesis (4, 5). Recently, some studies have shown that miRNAs are involved in synaptic transmission, plasticity, and cognition (6–9). Our previous study has demonstrated that miR-204-3p attenuates synaptic dysfunctions and oxidative stress in AD mice (10). MiR-431 plays an essential role in neurological disorders, including Parkinson’s disease, glioma, and spinal cord injury (11–13). Notably, miR-431 protects against synapse loss induced by amyloid-β (Aβ) in neuronal cells (14). However, whether miR-431 contributes to the pathogenesis of AD remains to be defined. Our microarray data have demonstrated that miR-431 is significantly reduced in the hippocampus of APPswe/PS1dE9 (APP/PS1) mice (10). In this study, we detected that miR-431 was remarkably decreased in the plasma of patients with amnestic mild cognitive impairment (aMCI) and AD. In addition, we found that miR-431 overexpression attenuated synaptic deficits and rescued memory deficits in APP/PS1 mice. Moreover, we showed that Smad4 was a direct target of miR-431, and inhibition of Smad4 protected against synaptic deficits in APP/PS1 mice, which suggested that miR-431/Smad4 might act as a potential therapeutic target for AD diagnosis and treatment.

## Results

### MiR-431 is decreased in the hippocampus and plasma of APP/PS1 mice and in the plasma of patients with aMCI and AD.

**Figure 1:**
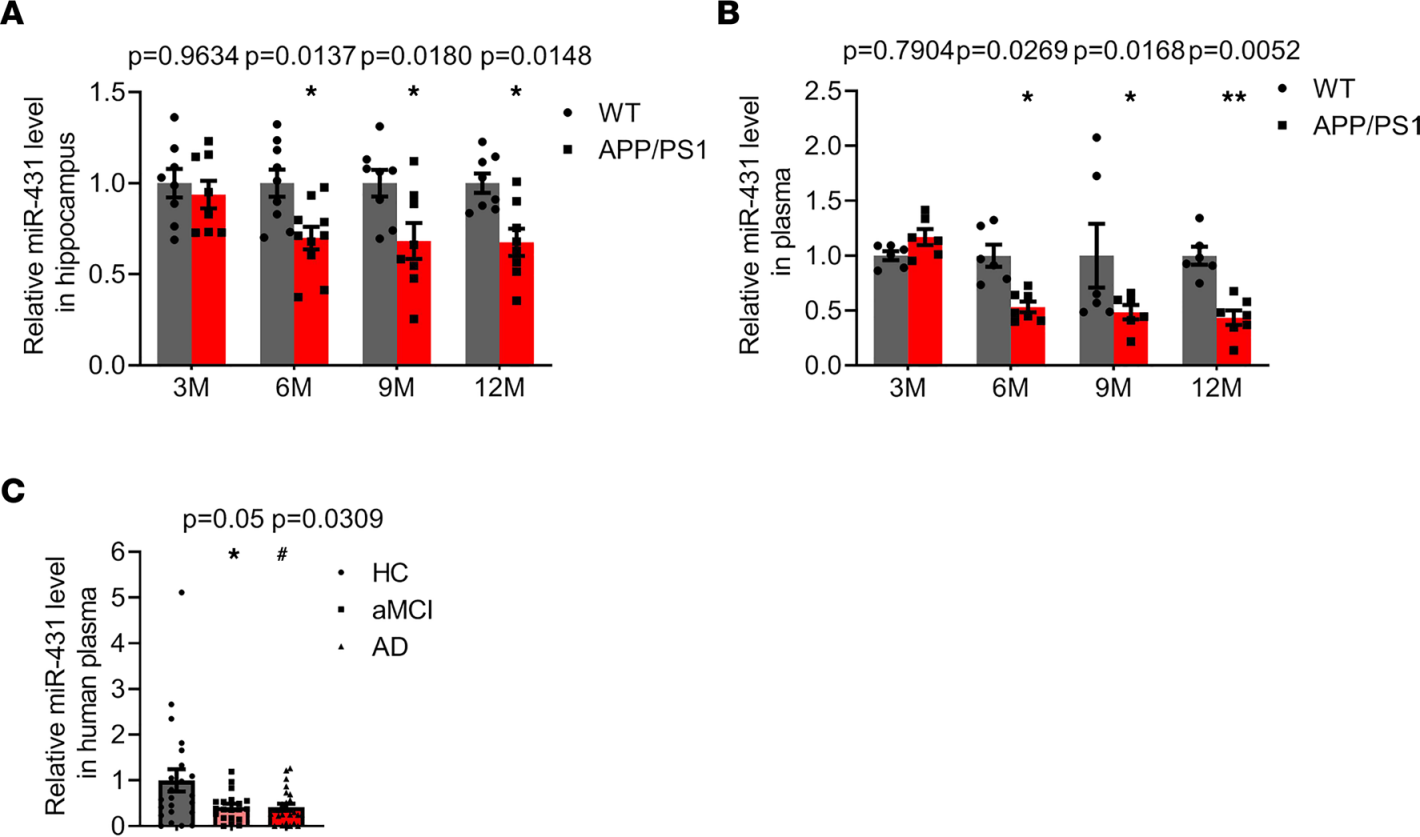
MiR-431 is downregulated in AD mice and patients with aMCI/AD. (**A**) The level of miR-431 was detected by real-time quantitative PCR (RT-qPCR) in the hippocampus of 3-, 6-, 9-, and 12-month-old APP/PS1 mice. *n* = 8–10 for each group. (**B**) The level of miR-431 was detected by RT-qPCR in the plasma of 3-, 6-, 9-, and 12-month-old WT and APP/PS1 mice. *n* = 6–7 for each group. *P < 0.05, **P < 0.01 vs. WT group. (**C**) The level of miR-431 in the plasma of AD patients (*n* = 25), aMCI patients (*n* = 20), and healthy controls (*n* = 23) was measured by RT-qPCR. *P < 0.05 vs. control group; ^#^*P* < 0.05 vs. control group. All data were presented as means ± SEM. Two-way ANOVA (**A** and **B**) and Kruskal-Wallis test followed by Dunn’s multiple comparison tests (**C**) were used.

MiR-431 was identified as a most decreased miRNA in the hippocampus of 6-month-old APP/PS1 mice by miRNA array (10). Here, we detected the expression of miR-431 in the hippocampus of APP/PS1 mice by quantitative PCR (qPCR) and found that it was downregulated in 6-, 9-, and 12-month-old APP/PS1 mice (Figure 1A). In addition, the level of miR-431 was reduced in the plasma of in 6-, 9-, and 12-month-old APP/PS1 mice (Figure 1B). Similarly, it was decreased in the plasma of patients with aMCI and AD (Figure 1C and Supplemental Table 1; supplemental material available online with this article; https://doi.org/10.1172/jci.insight.166270DS1), indicating that miR-431 might be an alternative biomarker for AD diagnosis.

### MiR-431 attenuates memory deficits in 6-month-old APP/PS1 mice.

**Figure 2:**
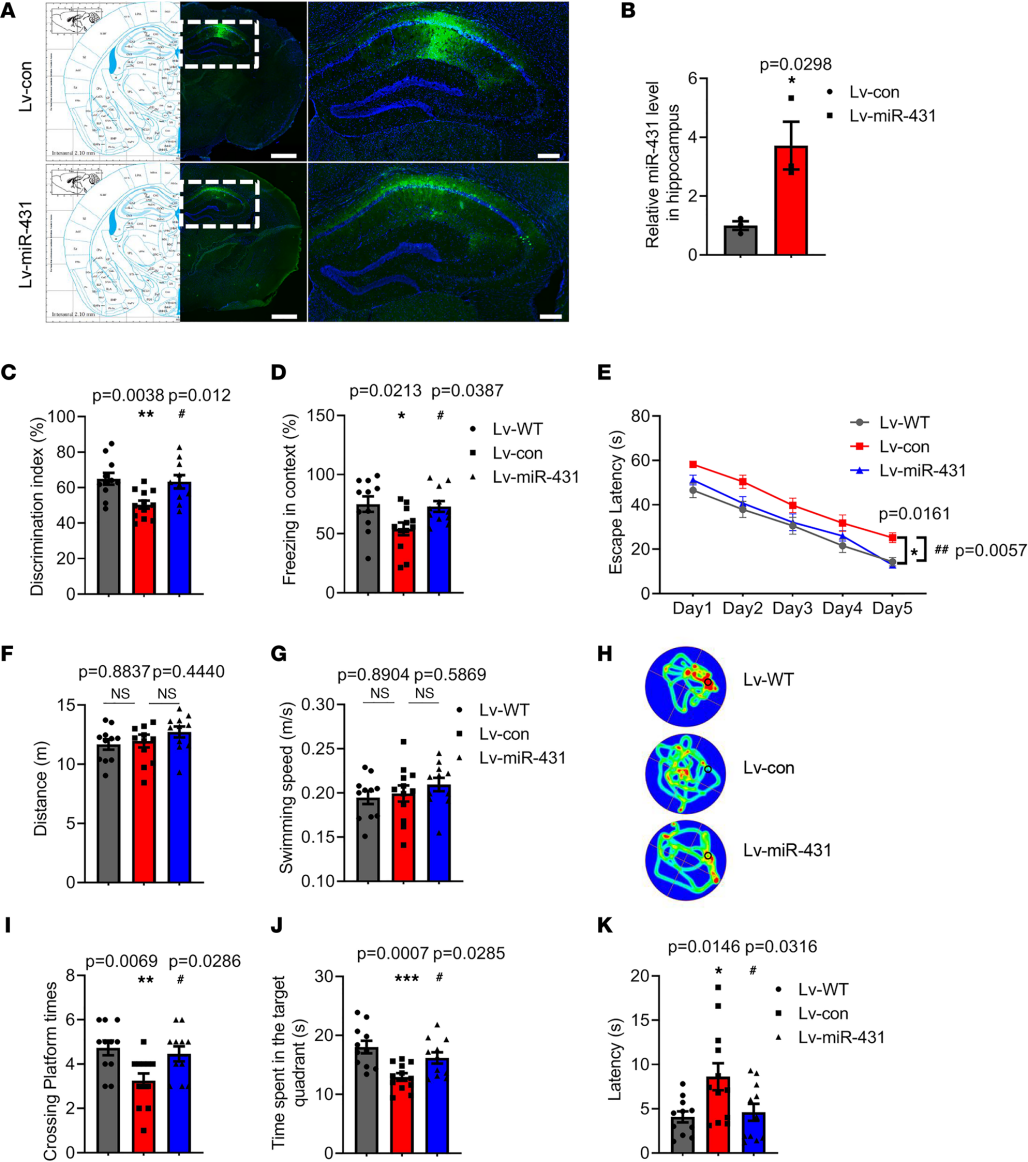
MiR-431 attenuates memory deficits in 6-month-old APP/PS1 mice. (**A**) A representative fluorescence image of Lv-con–infected and Lv-miR-431–infected slice. Left panel, bar = 1,000 μm; right panel, bar = 200 μm. (**B**) The expression of miR-431 was detected by RT-qPCR in the hippocampus after Lv-miR-431 treatment. *n* = 3 for each group. (**C**) The time exploring the objects was recorded by a visual tracking system in the NOR tests. *F* (2, 31) = 6.927, *P* = 0.0034. (**D**) The freezing time in contextual FC tests were recorded. *F* (2, 31) = 4.478, *P* = 0.0196. The escape latency (**E**) [groups: *F* (4, 155) = 65.63, *P* < 0.0001; days: *F* (2, 155) = 20.02, *P* < 0.0001; group × day: *F* (8, 155) = 0.2834, *P* = 0.9707] was detected in the acquisition trial. Distance (**F**) [*F* (2, 31) = 0.2138, *P* = 0.8087], swimming speed (**G**) [*F* (2, 31) = 0.8364, *P* = 0.4428], representative swimming paths (**H**), crossing platform times (**I**) [*F* (2, 31) = 5.688, *P* = 0.0079], time spent in target quadrant (**J**) [*F* (2, 31) = 8.398, *P* = 0.0012] and latency to target quadrant (**K**) [*F* (2, 31) = 4.937, *P* = 0.0138] were recorded in the probe trial in the MWM tests. *n* = 11–12 for each group. *P < 0.05, **P < 0.01, ***P < 0.001 vs. Lv-WT group; ^#^*P* < 0.05, ^##^*P* < 0.01 vs. Lv-con group. All data were presented as means ± SEM. Two-tailed unpaired Student’s *t* test (**B**), 1-way ANOVA (**C**, **D**, **F**, **G**, and **I**–**K**), and 2-way ANOVA (**E**) were used.

To determine the role of miR-431 on memory functions in APP/PS1 mice, a miR-431 overexpression lentivirus (Lv-miR-431) and a control lentivirus (Lv-con) were constructed and injected into bilateral hippocampus CA1 of 6-month-old APP/PS1 mice as demonstrated by fluorescence microscopy (Figure 2A). The level of miR-431 was significantly increased in the hippocampus of Lv-miR-431–treated APP/PS1 mice compared with that in the Lv-con group (Figure 2B). The term Lv-WT refers to wild-type mice treated with Lv-con by stereotaxic apparatus in the results and figures. Behavior tests including open field (OF) tests, novel object recognition (NOR) tests, Morris water maze (MWM) tests, and contextual fear conditioning (FC) tests were performed. In the OF tests, miR-431 overexpression did not alter the distance and the time spent in corner and center areas (Supplemental Figure 1, A–C), suggesting that miR-431 does not modulate the motor function and emotional state of APP/PS1 mice. In addition, miR-431 overexpression significantly increased the discrimination index in NOR tests (Figure 2C) and the freezing time in contextual FC tests (Figure 2D) of APP/PS1 mice. In the MWM tests, miR-431 overexpression significantly decreased the escape latency of APP/PS1 mice in the acquisition trial (Figure 2E). Furthermore, in the probe trial, miR-431 overexpression did not affect the total distance (Figure 2F) and the swimming speed (Figure 2G). In addition, the crossing platform times (Figure 2, H and I) and the time spent in the target quadrant were notably decreased (Figure 2J), while the latency time to the target quadrant was also decreased in Lv-miR-431–treated APP/PS1 mice (Figure 2K). Collectively, these results suggested that miR-431 overexpression attenuates contextual fear memory and spatial memory deficits in APP/PS1 mice.

### MiR-431 overexpression barely affects Aβ levels in the hippocampus of 6-month-old APP/PS1 mice.

**Figure 3:**
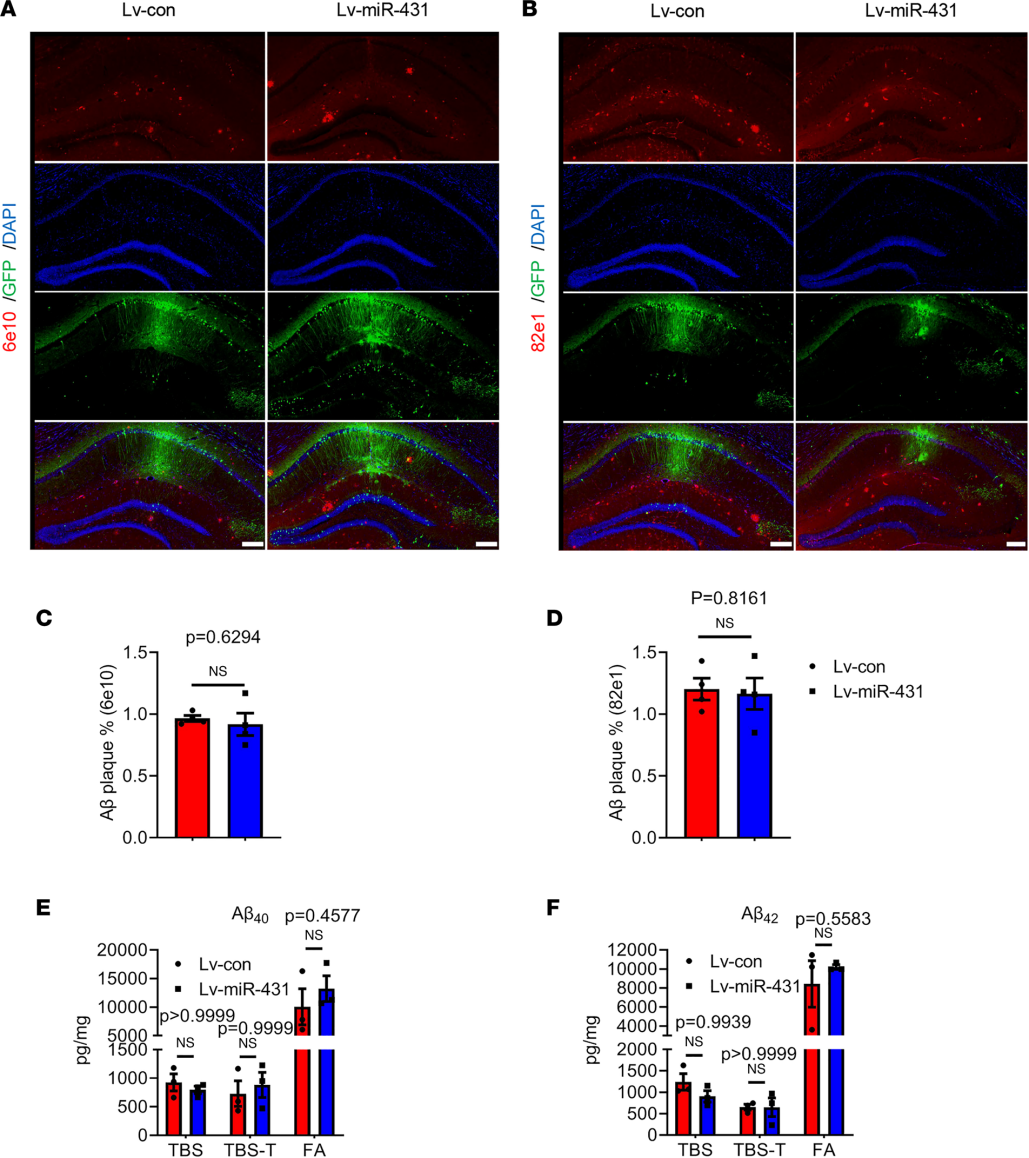
MiR-431 does not affect Aβ levels in the hippocampus of 6-month-old APP/PS1 mice. (**A**) The level of Aβ 6e10 in the hippocampus of Lv-miR-431–treated APP/PS1 mice was detected by immunofluorescence staining. (**B**) The level of Aβ 82e1 in the hippocampus of Lv-miR-431–treated APP/PS1 mice. Bars = 100 μm. (**C**) Quantitative analysis of the percentage of Aβ 6e10–positive area. *n* = 4 mice per group. Bar = 200 μm. (**D**) Quantitative analysis of the percentage of Aβ 82e1–positive area. *n* = 4 mice per group. Bar = 200 μm. The protein levels of TBS-soluble, TBS-T–soluble, and FA-soluble Aβ_40_ (**E**) and Aβ_42_ (**F**) were measured by ELISA in the hippocampus of Lv-miR-431–treated APP/PS1 mice. *n* = 3, NS. All data were presented as means ± SEM. Two-tailed unpaired Student’s *t* test (**C** and **D**) and 2-way ANOVA (**E** and **F**) were used. TBS-T, TBS-Tween; FA, formic acid.

Given that Aβ is one of the key pathological features of AD and Aβ clearance has promising therapeutic effects on AD (15), we determined whether miR-431 decreased the Aβ levels in the brains of APP/PS1 mice. As shown in Figure 3, A–D, the Aβ load (6e10 and 82e1) was not significantly changed in the hippocampus of Lv-miR-431–treated APP/PS1 mice. In addition, miR-431 overexpression did not affect the soluble and insoluble Aβ_40/42_ in the hippocampus of APP/PS1 mice (Figure 3, E and F). These data indicated that miR-431 might enhance the memory functions in an Aβ-independent pathway.

### MiR-431 overexpression improves synaptic plasticity in 6-month-old APP/PS1 mice.

**Figure 4:**
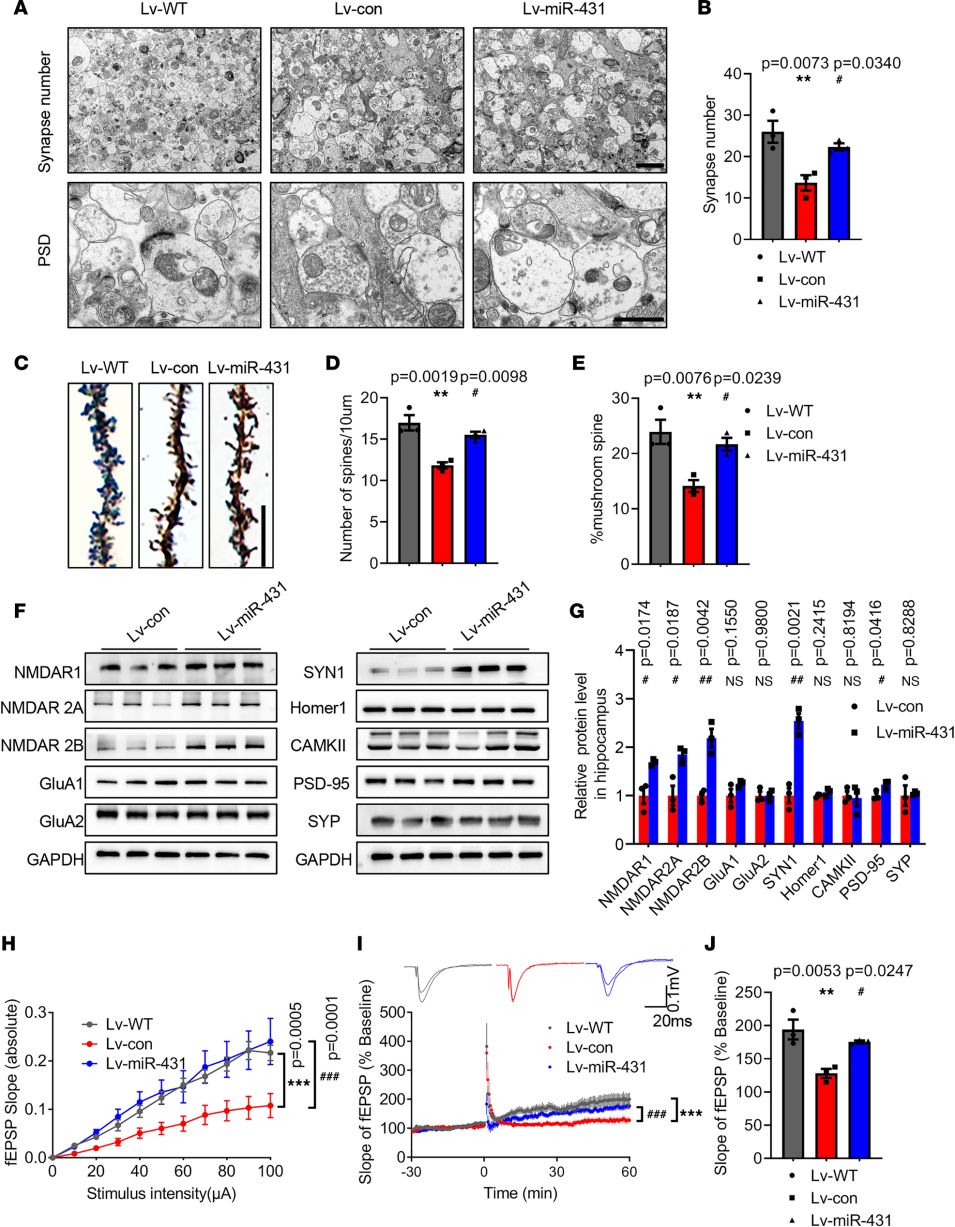
MiR-431 overexpression improves synaptic plasticity in 6-month-old APP/PS1 mice. (**A**) Electron micrographs displaying synapse numbers (upper panel, bar = 2 μm) and the thickness of PSD (lower panel, bar = 1 μm) in the hippocampus CA1 after Lv-miR-431 treatment. (**B**) Quantitative analysis of synapse numbers. *n* = 3 mice per group. *F* (2, 6) = 10.72, *P* = 0.0104. (**C**) Golgi staining in the hippocampus CA1 after Lv-miR-431 treatment. Bar = 10 μm. Quantification of spine density (**D**) [*F* (2, 6) = 18.58, *P* = 0.0027] and mushroom spines percentage (**E**) [*F* (2, 6) = 10.99, *P* = 0.0099], *n* = 3 mice per group. (**F** and **G**) The expressions of synaptic proteins were measured by Western blot. (**H**) The input/output (I/O) slope of hippocampal CA1 in Lv-miR-431–treated APP/PS1 mice. (**I** and **J**) High-frequency induced LTP stimulation was observed in hippocampal CA1 area. *F* (2, 6) = 12.85, *P* = 0.0068. *n* = 3 mice per group. **P < 0.01, ***P < 0.001 vs. Lv-WT group; ^#^*P* < 0.05, ^##^*P* < 0.01, ^###^*P* < 0.001 vs. Lv-con group. All data were presented as means ± SEM. One-way ANOVA (**B**, **D**, **E**, **I**, and **J**), 2-tailed unpaired Student’s *t* test (**G**), and 2-way ANOVA (**H**) were used.

Since synaptic plasticity is considered the basis of memory functions (16, 17), next we determined the effects of miR-431 on the synaptic structure and synaptic transmission. The results of electron micrographs showed that the number of synapses and the thickness of postsynaptic density (PSD) were remarkably decreased in the CA1 area of APP/PS1 mice, and miR-431 overexpression partially rescued these pathological features (Figure 4, A and B). Meanwhile, miR-431 increased the dendritic spine density and the percentage of mushroom spines in the CA1 area of APP/PS1 mice (Figure 4, C–E). In addition, miR-431 overexpression increased the levels of synaptic proteins including synapsin1 (SYN1), postsynaptic density protein 95 (PSD-95), NMDAR subunit 1 (NMDAR1), NMDAR2A, and NMDAR2B in the hippocampus of APP/PS1 mice, whereas the levels of Homer1, a-amino-3-hydroxy-5-methyl-4-isoxazole propionate (AMPA) receptor subunit GluA1, GluA2, and calcium/calmodulin-dependent protein kinase II (CaMKII) were not significantly changed (Figure 4, F and G). To explore the effect of miR-431 on synaptic transmission, we examined the long-term potentiation (LTP) in hippocampal neurons of APP/PS1 mice, and the results showed miR-431 significantly increased the slope of hippocampus slices and LTP magnitudes (Figure 4, H–J). These results suggested that miR-431 overexpression mitigated synaptic plasticity deficits in APP/PS1 mice. Furthermore, similar results were obtained in the in vitro experiments. The synaptic density and the PSD-95 and SYN1 protein levels were decreased in hippocampal neurons of APP/PS1 mice compared with in WT mice, and miR-431 overexpression significantly increased the synaptic density (Supplemental Figure 2, A–D) and the PSD-95 and SYN1 protein levels (Supplemental Figure 2, E–G).

### MiR-431 is a target of Smad4.

**Figure 5:**
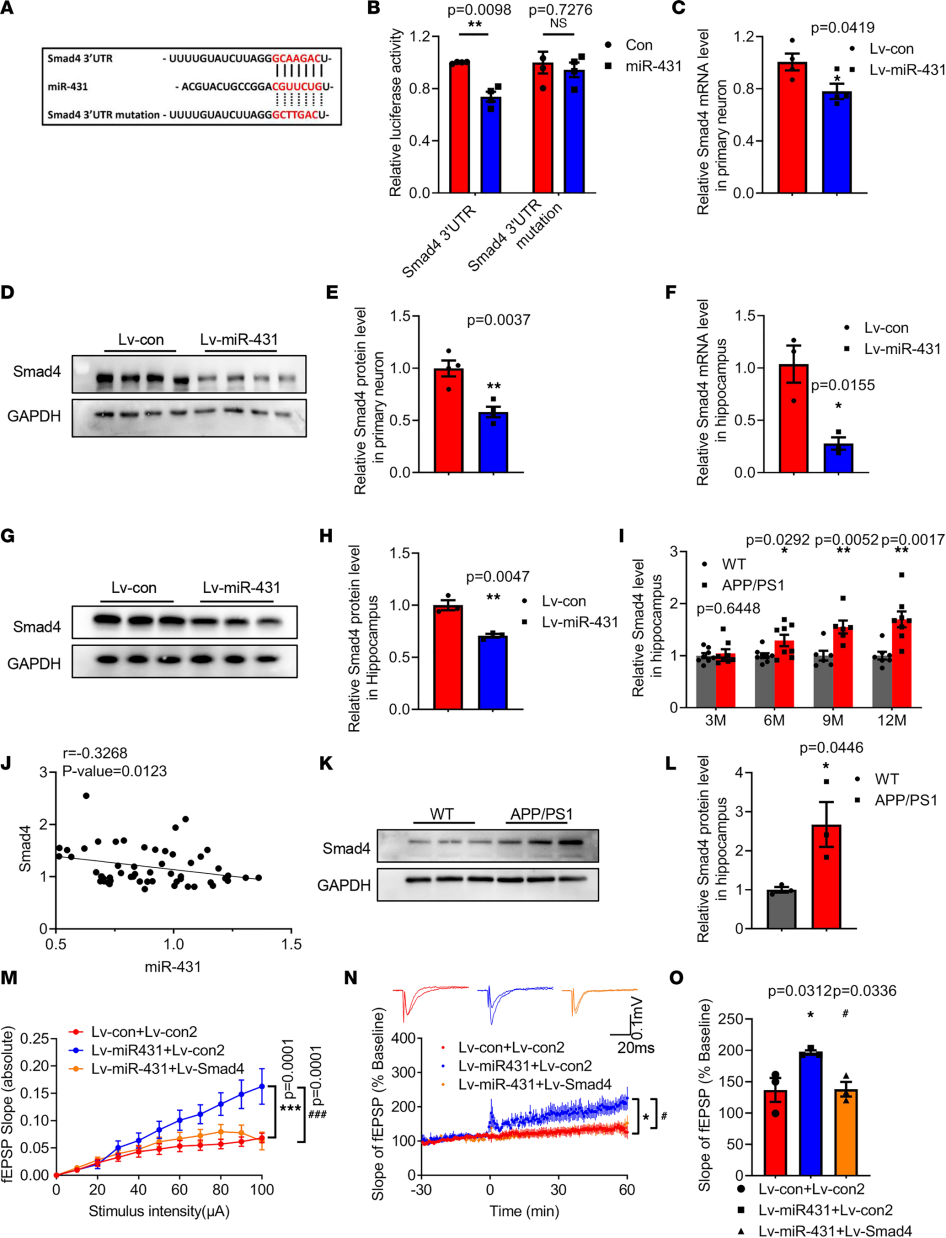
Smad4 is a target of miR-431. (**A**) TargetScan prediction of the binding sites of miR-431 with Smad4 3′-UTR. (**B**) HEK293T cells were infected by Lv-miR-431 for 72 hours followed by transfecting with Smad4 3′-UTR or mutant Smad4 3′-UTR for another 24 hours, and relative luciferase activity was determined. *n* = 4. The mRNA (**C**) and protein levels (**D** and **E**) of Smad4 in Lv-miR-431–treated primary neurons. *n* = 4. The mRNA (**F**) and protein (**G** and **H**) levels of Smad4 in the hippocampus after Lv-miR-431 treatment. *n* = 3. *P < 0.05, **P < 0.01 vs. Lv-con group. (**I**) The mRNA level of Smad4 in the hippocampus of 3-, 6-, 9-, and 12-month-old APP/PS1 mice. *n* = 6–8 for each group. (**J**) The expression correlation of miR-431 and Smad4 mRNA level in WT and APP/PS1 mice as shown in panel. *n* = 58. (**K** and **L**) The protein level of Smad4 in the hippocampus of 6-month-old WT and APP/PS1 mice. *n* = 3. *P < 0.05, **P < 0.01 vs. WT group. (**M**) The I/O slope of hippocampal CA1 in Lv-miR-431– and Lv-Smad4–treated APP/PS1 mice. (**N** and **O**) High-frequency induced LTP stimulation was observed in hippocampal CA1 area. *F* (2, 6) = 6.858, *P* = 0.0282, *n* = 3 mice per group. *P < 0.05, ***P < 0.001 vs. Lv-con+Lv-con2 group; ^#^*P* < 0.05, ^###^*P* < 0.001 vs. Lv-miR-431+Lv-con2 group. All data were presented as means ± SEM. Two-way ANOVA (**B**, **I**, and **M**), 2-tailed unpaired Student’s *t* test (**C**, **E**, **F**, **H**, and **L**), Pearson’s correlation (**J**), and 1-way ANOVA (**N** and **O**) were used.

There was a potential binding site in Smad4 3′-UTR with miR-431 as predicted by TargetScan (Figure 5A). The results of luciferase reporter assay showed that miR-431 overexpression significantly inhibited the relative luciferase units (RLUs) of Smad4 3′-UTR, while it failed to affect the RLUs of Smad4 3′-UTR mutant (Figure 5B). These results indicated that miR-431 specifically bound with Smad4 3′-UTR. To confirm Smad4 as a target of miR-431, we detected the mRNA and protein levels of Smad4 in miR-431 overexpressed primary neurons and hippocampus. The results showed the mRNA (Figure 5C) and protein (Figure 5, D and E) levels of Smad4 were significantly decreased in Lv-miR-431–treated neurons, and similar results were observed in the hippocampus of Lv-miR-431–treated APP/PS1 mice (Figure 5, F–H), suggesting that miR-431 inhibited the level of Smad4 by specifically binding with its 3′-UTR.

The mRNA level of Smad4 was significantly increased in the hippocampus of 6-, 9-, and 12-month-old APP/PS1 mice, and it was negatively correlated with the level of miR-431 (Figure 5, I and J). The protein level of Smad4 was also increased in the hippocampus of 6-month-old APP/PS1 mice (Figure 5, K and L). To further determine whether Smad4 inhibition mediated the protective effects of miR-431, we measured the LTP in hippocampal neurons of APP/PS1 mice treated with Lv-miR-431 and a lentivirus overexpressing Smad4 (Lv-Smad4). The overexpression efficiency of Lv-Smad4 was verified in Supplemental Figure 3. The results showed that miR-431 overexpression significantly increased the slope of hippocampus slices and LTP magnitudes, while overexpression of Smad4 abolished these effects, suggesting that miR-431 enhanced synaptic plasticity at least partially by inhibition of Smad4 (Figure 5, M–O).

### Smad4 inhibition alleviates memory deficits in 6-month-old APP/PS1 mice.

**Figure 6:**
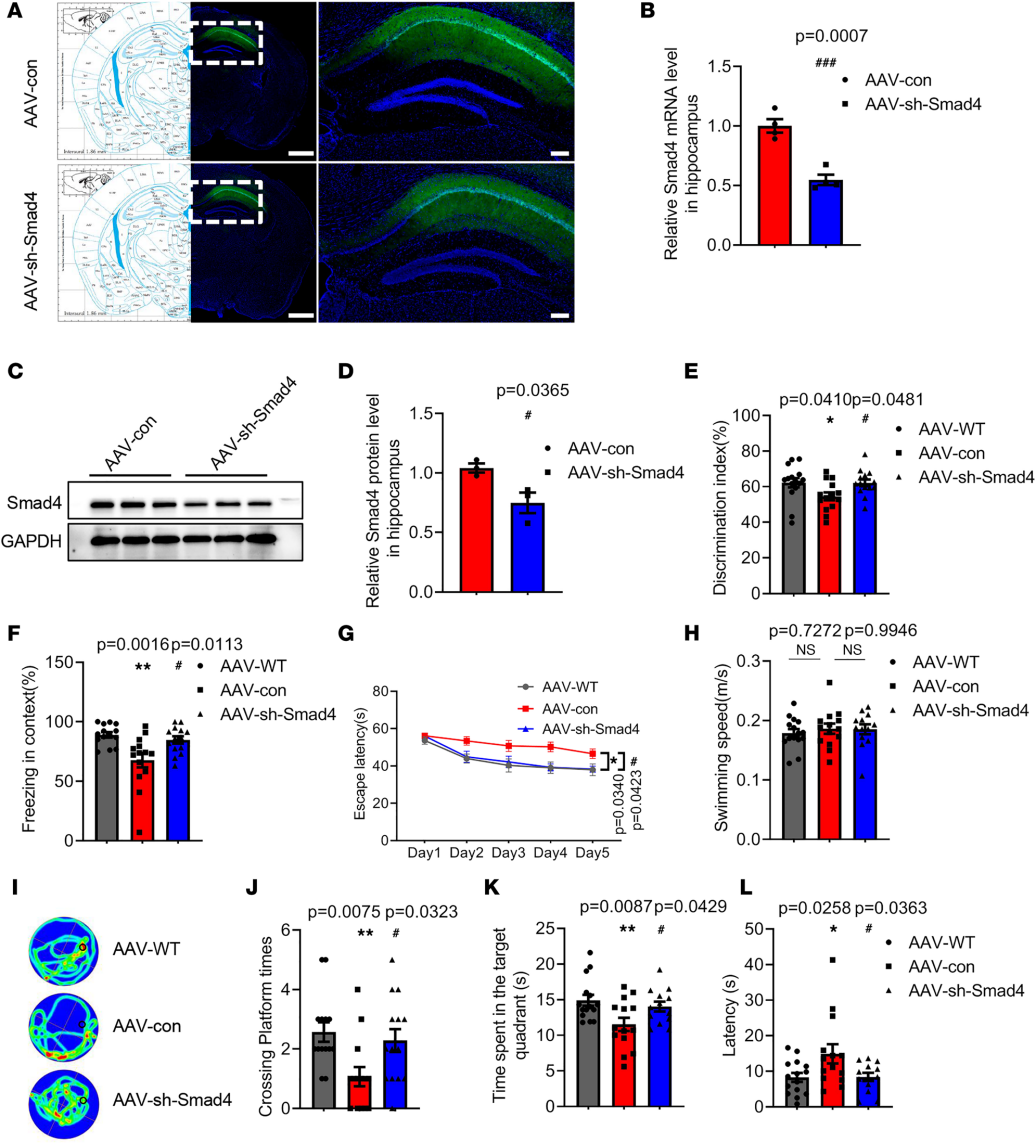
Smad4 inhibition alleviates memory deficits in 6-month-old APP/PS1 mice. (**A**) A representative fluorescence image of the AAV-con-infected and AAV-sh-Smad4-infected slice. Left panel, bar = 1,000 μm, right panel, bar = 200 μm. The mRNA (**B**) and protein (**C** and **D**) levels of Smad4 in the hippocampus after AAV-sh-Smad4 treatment. *n* = 4 for mRNA level and *n* = 3 for protein level. (**E**) The time exploring the objects was recorded by a visual tracking system in the NOR tests. *F* (2, 39) = 3.644, *P* = 0.0352. (**F**) The freezing time in contextual FC tests was recorded. *F* (2, 39) = 7.391, *P* = 0.0019. Escape latency (**G**) [groups: *F* (4, 195) = 14.62, *P* < 0.0001; days: *F* (2, 195) = 16.16, *P* < 0.0001; group × day: *F* (8, 195) = 0.7725, *P* = 0.6274] was detected in the acquisition trial. The swimming speed (**H**) [*F* (2, 39) = 0.2675, *P* = 0.7667], representative swimming paths (**I**), crossing platform times (**J**) [*F* (2, 39) = 5.311, *P* = 0.0091], time spent in target quadrant (**K**) [*F* (2, 39) = 4.811, *P* = 0.0136], and latency to target quadrant (**L**) [*F* (2, 39) = 4.203, *P* = 0.0222] were recorded in the probe trial in the MWM tests. *n* = 14. *P < 0.05, **P < 0.01 vs. AAV-WT group; ^#^*P* < 0.05, ^###^*P* < 0.001 vs. AAV-con group. All data were presented as means ± SEM. Two-tailed unpaired Student’s *t* test (**B** and **D**), 1-way ANOVA (**E**, **F**, **H**, and **J**–**L**), and 2-way ANOVA (**G**) were used.

To explore the effect of Smad4 on memory function, Smad4 was knocked down by stereotactic injection of an adeno-associated virus (AAV-sh-Smad4) in bilateral hippocampus CA1 of 6-month-old APP/PS1 mice, and the behavior tests were performed. The EGFP-tagged AAV-con and AAV-sh-Smad4 were extensively expressed (Figure 6A), and the mRNA (Figure 6B) and protein (Figure 6, C and D) levels of Smad4 were significantly reduced in the hippocampus of AAV-sh-Smad4–treated APP/PS1 mice. The term AAV-WT refers to wild-type mice injected with control AAV by stereotaxic apparatus in the results and figures. The results of OF tests showed that inhibition of Smad4 had no effect on the distance traveled or the time spent in corner and center areas (Supplemental Figure 4, A–C). However, Smad4 inhibition significantly increased the discrimination index in the NOR tests (Figure 6E) and the freezing time in contextual FC tests (Figure 6F) of APP/PS1 mice. In the MWM tests, Smad4 inhibition significantly decreased the escape latency in the acquisition tests (Figure 6G). Moreover, in the probe trial, total distance (Supplemental Figure 4D) and swimming speed (Figure 6H) were not affected by Smad4 inhibition. Smad4 inhibition increased the crossing platform times (Figure 6, I and J), as well as the time spent in the target quadrant (Figure 6K), and decreased the latency to arrive at the target quadrant (Figure 6L). Collectively, these results indicated that Smad4 inhibition partially rescues the memory deficits in APP/PS1 mice.

### Smad4 inhibition attenuates synaptic plasticity deficits in 6-month-old APP/PS1 mice.

**Figure 7:**
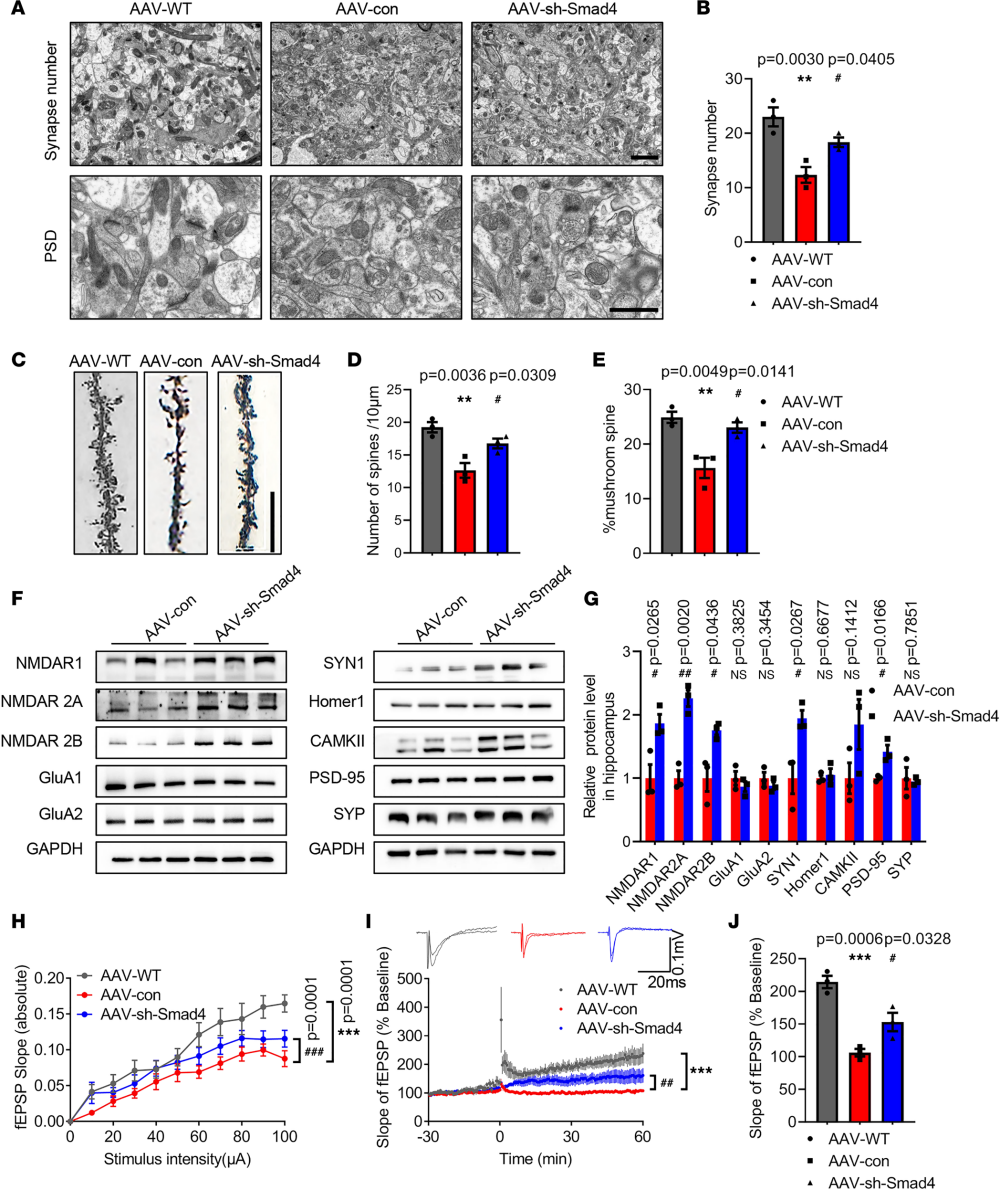
Inhibition of Smad4 attenuates synaptic plasticity deficits in 6-month-old APP/PS1 mice. (**A**) Electron micrographs displaying synapse numbers (upper panel, bar = 1 μm) and the thickness of PSD (lower panel, bar = 2 μm) in the hippocampus CA1 after AAV-sh-Smad4 treatment. (**B**) Quantitative analysis of synapse numbers. *n* = 3 mice per group. *F* (2, 6) = 14.57, *P* = 0.0050. (**C**) Golgi staining in the hippocampus CA1 after AAV-sh-Smad4 treatment. Bar = 10 μm. Quantification of spine density (**D**) [*F* (2, 6) = 13.78, *P* = 0.0057] and mushroom spine percentage (**E**) [*F* (2, 6) = 13.38, *P* = 0.0061]. *n* = 3 mice per group. (**F** and **G**) The expressions of synaptic proteins were measured by Western blot. (**H**) The I/O slope of hippocampal CA1 in Lv-miR-431–treated APP/PS1 mice. (**I** and **J**) High-frequency induced LTP stimulation was observed in hippocampal CA1 area. *F* (2, 6) = 27.32, *P* = 0.0010. *n* = 3 mice per group. **P < 0.01, ***P < 0.001 vs. AAV-WT group; ^#^*P* <0.05, ^##^*P* < 0.01, ^###^*P* < 0.001 vs. AAV-con group. All data were presented as means ± SEM. One-way ANOVA (**B**, **D**, **E**, **I**, and **J**), 2-tailed unpaired Student’s *t* test (**G**), and 2-way ANOVA (**H**) were used.

Accordingly, the load of Aβ plaques and soluble/insoluble Aβ_40/42_ in the hippocampus of APP/PS1 mice remained unchanged upon Smad4 inhibition (Supplemental Figure 5). To determine whether Smad4 inhibition improved synaptic functions in APP/PS1, we measured the synaptic numbers, morphology, synaptic transmission, and synaptic related proteins. The results of electron micrographs showed that the number of synapses and thickness of PSD in the CA1 area in the AAV-con group were notably decreased compared with in AAV-WT mice. Additionally, Smad4 inhibition significantly increased the number of synapses and thickness of PSD in APP/PS1 mice (Figure 7, A and B). The dendritic spine density and the percentage of mushroom spines in CA1 area in AAV-con group were significantly decreased compared with those in AAV-WT group. Moreover, Smad4 inhibition increased the dendritic spine density and the percentage of mushroom spines in APP/PS1 mice as demonstrated by Golgi staining (Figure 7, C–E). Inhibition of Smad4 also increased the protein levels of SYN1, PSD-95, NMDAR1, NMDAR2A, and NMDAR2B in the hippocampus of APP/PS1 mice (Figure 7, F and G). Furthermore, inhibition of Smad4 significantly increased the slope of hippocampus slices and LTP magnitudes (Figure 7, H–J). These results suggested Smad4 inhibition improves both synaptic structure and function in the hippocampus CA1 of APP/PS1 mice.

### Smad4 negatively regulates the expression of Dlg3 by directly binding to –1,049 to –776 bp of the Dlg3 promoter.

**Figure 8:**
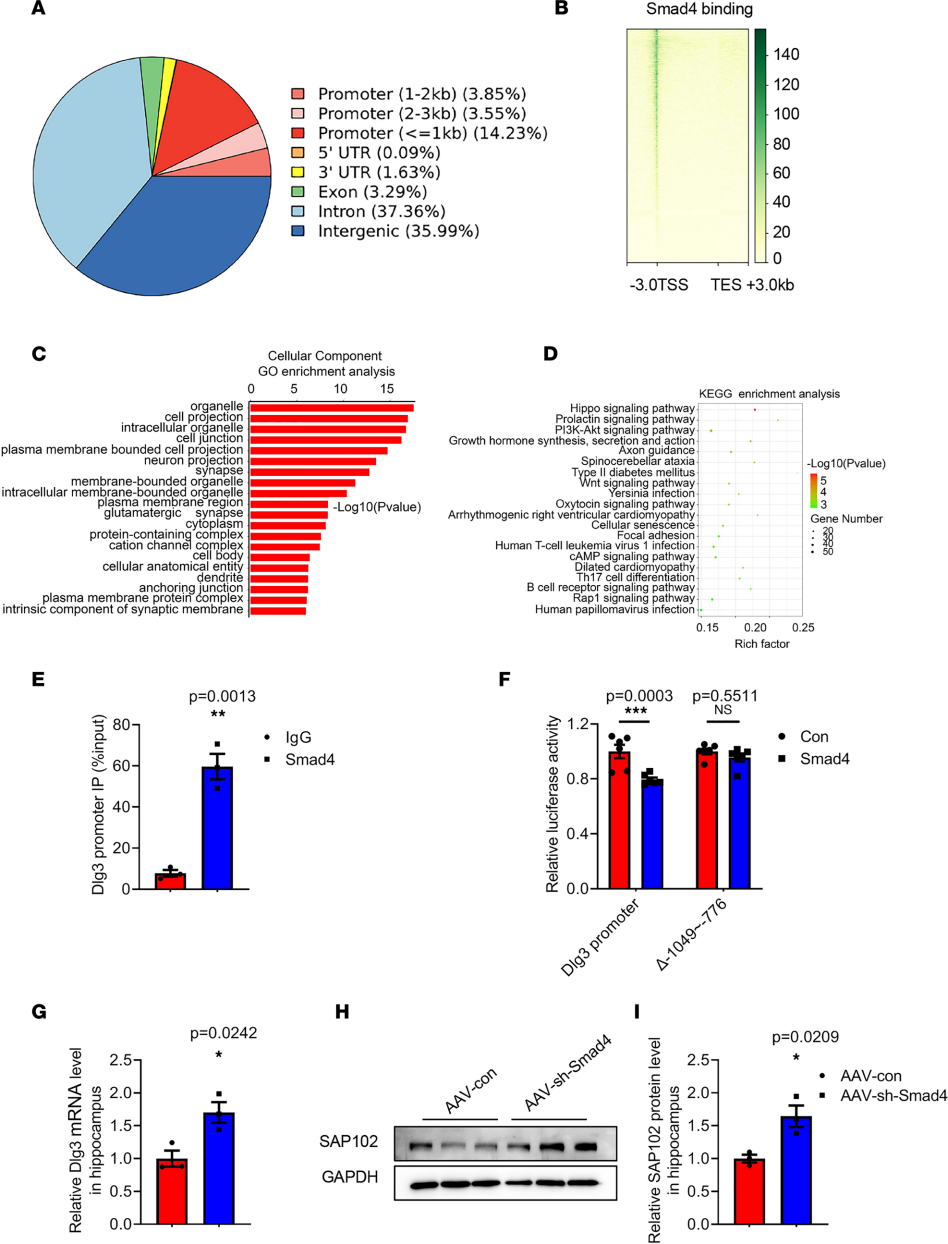
Smad4 inhibits the expression of Dlg3 by directly binding to –1,049 to –776 bp in the promoter. (**A**) The genomic distribution of Cut&Tag peaks. (**B**) Smad4 gene-binding pattern. Smad4 bound with the TSS. (**C**) Cellular component GO enrichment analysis. (**D**) KEGG enrichment analysis. (**E**) ChIP analysis was performed to assess the interaction of Smad4 and the Dlg3 promoter. *n* = 3 per group. **P < 0.01 vs. IgG group. (**F**) Luciferase activity in HEK293T cells. *n* = 3 for each group. Control group (con) vs. Smad4 overexpression group (Smad4), Dlg3 promoter, ***P < 0.001; truncated Dlg3 promoter (Δ–1049~–776 bp), NS. The mRNA (**G**) and protein (**H** and **I**) levels of Dlg3/SAP102 in the hippocampus of AAV-sh-Smad4–treated APP/PS1 mice. *n* = 3. *P < 0.05 vs. AAV-con group. All data were presented as means ± SEM. Two-tailed unpaired Student’s *t* test (**E**, **G**, and **I**) and 2-way ANOVA (**F**) were used.

Smad4 forms a complex with other phosphorylated regulatory Smads and translocates into the nucleus to regulate gene transcription (18). A chromosome Cleavage Under Targets and Tagmentation (Cut&Tag) assay was performed to explore the potential targets of Smad4, and it showed that a total of 5,327 annotated genome-binding peaks were identified and that Smad4 bound with the transcription start sites (TSSs) of 1,152 target genes (Figure 8, A and B). Gene Ontology (GO) enrichment analysis of these target genes showed that multiple genes, including discs large MAGUK scaffold protein 3 (Dlg3), microtubule-associated protein 1A, glutamate ionotropic receptor NMDA type subunit 2C, and Neurexin1, were enriched in synapses (Figure 8C). Kyoto Encyclopedia of Genes and Genomes (KEGG) enrichment analysis of the target genes demonstrated that several pathways, including Axon guidance and Hippo signaling pathway, were associated with synaptic functions (Figure 8D). Smad4 potentially bound to the segment approximately –1,049 to –776 bp from the ATG start codon of Dlg3, which was verified by the chromatin immunoprecipitation (ChIP) assay (Figure 8E). To examine whether Smad4 modulated Dlg3 expression through interaction with the segment –1,049 to –776 bp, the Dlg3 promoter (–2,500 ~+2 bp) and its truncated form (–1,049 to –776 bp deleted) were constructed. The results showed that Smad4 overexpression inhibited the transcriptional activity of Dlg3, which was abolished by the truncated form (–1,049 to –776 bp deleted) (Figure 8F). Furthermore, both Dlg3 mRNA and protein (SAP102) levels were increased in the hippocampus of AAV-sh-Smad4–treated mice (Figure 8, G–I). Collectively, our data demonstrated that Smad4 negatively affected the expression of Dlg3 by directly binding to the –1,049 to –776 bp of the Dlg3 promoter. However, further studies are needed to investigate whether the protective effects of Smad4 inhibition on synaptic plasticity in APP/PS1 mice are mediated by Dlg3 induction.

## Discussion

In this study, we have demonstrated a key role of miR-431 on the synaptic plasticity and memory functions in APP/PS1 mice. The miR-431 level was decreased in the hippocampus and plasma of 6-, 9-, and 12-month-old APP/PS1 mice. Notably, the level of miR-431 was also downregulated in the plasma of patients with aMCI and AD. MiR-431 overexpression in the hippocampus CA1 ameliorated synaptic and memory dysfunction of AD mice. In addition, Smad4 was identified as a direct target of miR-431, and Smad4 knockdown showed a protective effect by enhancing synaptic plasticity and memory functions in APP/PS1 mice. Moreover, Smad4 overexpression reversed the protective effects of miR-431 and inhibited the expression of Dlg3 by directly binding to –1,049 to –776 bp of the Dlg3 promoter. Therefore, our data indicated that miR-431/Smad4 might be a potential biomarker and therapeutic target for AD.

Synaptic plasticity impairment has been considered as an early hallmark in the pathogenesis of AD, which may emerge before clinical symptoms and the deposition of Aβ plaques and p-tau tangles in the brain (19). Synaptic dysfunctions disrupt neural circuits, cause extensive neural network abnormalities and cognitive decline, and aggravate the progress of neurodegeneration (20, 21). The number of synapses in the inferior temporal gyrus, hippocampal CA1 area, dentate gyrus, and posterior cingulate gyrus in the early stage of AD is significantly reduced compared with in individuals with normal cognitive function. In addition, the cognitive impairment is closely related to synaptic density in these patients with AD (22, 23). Recently, it was shown that postsynaptic targets and synaptic shapes are altered in the early stage of AD, while synaptic density and morphological alterations of the remaining synapses are severely impaired in late stages of AD using focused ion beam/scanning electron microscopy (24). Synaptic proteins including PSD-95, synaptophysin, and AMPA and NMDA receptors contribute to synaptic transmission and functions (25–27). LTP is generally compromised in Aβ-treated hippocampal slices and AD mice, and enhancing LTP may preserve synaptic plasticity and memory functions (22, 28, 29). Here, we have shown that miR-431 overexpression or Smad4 knockdown attenuates synaptic dysfunctions and memory impairment in APP/PS1 mice, while it does not affect the Aβ levels in the brain, which indicates that miR-431/Smad4 modulates memory functions in an Aβ-independent pathway.

MiRNAs regulate the biological functions of synaptic plasticity and neurogenesis and play important roles in neurodegenerative diseases (30–34). The expressions of miR-26b, miR-34, and miR-125b are significantly increased, while miR-9, miR-29a, and miR-106 are decreased, in cerebrospinal fluid (CSF) and plasma of patients with AD (35). MiR-132 is significantly downregulated in AD, and miR-132 restoring facilitates the expression of postsynaptic membrane glutamatergic receptors and the number and morphology of spines (36, 37). In addition, our previous study shows that miR-204-3p alleviates memory impairment by mitigating synaptic dysfunctions and oxidative stress in APP/PS1 mice (10). MiR-431 is extensively expressed in the central nervous system and has participated in the pathogenesis of neurological disorders. MiR-431 overexpression affects the length of motor nerve processes and reverses the symptoms of muscle atrophy (38, 39). Furthermore, in an in vitro AD model, miR-431 reduces the degeneration of membrane neuritis and the loss of synapses induced by Aβ_1–42_ by inhibition of Dickkopf-1 (14). In this study, it was shown that the level of miR-431 was reduced in the hippocampus and plasma in AD mice, and hippocampus CA1–specific overexpression of miR-431 significantly increased spine density, synaptic structures, and synaptic proteins and enhanced synaptic transmission, which eventually improved the memory functions of APP/PS1 mice. Since a ubiquitous promoter-based lentivirus was used in this study, our results did not exclude the effect of miR-431 in non-neuronal cells on the pathogenesis of AD, which was a limitation of our study.

Smad4, a central intracellular signal transmission mediator of transmission of TGF-β signaling, plays a pivotal role in many biological processes, including cell differentiation, migration, apoptosis, and tumorigenesis (40, 41). Smad4 is highly expressed in the central nervous system and regulates the balance between proliferation and differentiation of neural stem cells (NSCs) in a spatiotemporal manner (42–44). In immature vomeronasal sensory neurons (VSNs), Smad4 loss of function compromises dendritic knob and glomeruli formation in the accessory olfactory bulb (AOB), but in mature VSNs, Smad4 loss of function only affects correct glomeruli formation in the AOB, indicating that Smad4-mediated signaling contributes to the functional maturation and connection of VSNs (45). It is reported that increased Smad4 drives the invasion pathways of glioma (46), and the level of Smad4 is lower in the dorsolateral prefrontal cortex and anterior cingulate cortex from individuals with schizophrenia compared with that in the control group (47). Emerging evidence has demonstrated that Smad4 is also involved in the pathogenesis of AD. Once TGF-β signaling is stimulated, Smad4 interaction with Sp1 and Smad3 induces the transcriptional activation of APP (48). Smad4 physically binds with TGF-β1–induced antiapoptotic factor (TIAF1) and prevents TIAF1 self-aggregation, which reduces production of Aβ and amyloid fibrils (49). Smad4 facilitates adult hippocampal APP/PS1 NSC differentiation to neurons and improves the cognitive ability of AD mice by interaction with Smad2/3 (50). Here, we demonstrated that Smad4 inhibition by miR-431 or AAV-mediated RNA interference strengthened the synaptic plasticity and ameliorated cognitive deficits in APP/PS1 mice. Interestingly, the immunofluorescence staining and ELISA results showed that the level of Aβ was not significantly affected in the hippocampus of AAV-sh-Smad4–treated mice. We speculated that in the in vivo AD models, Smad4 might be involved in a more complex interaction with other transcriptional factors, which led to the inconsistent effects on Aβ levels. The results of Cut&Tag assay showed Smad4 modulated multiple genes associated with synapse function. Dlg3 is extensively expressed in the postsynaptic densities of excitatory synapses and participates in receptor-mediated synaptic transmission by binding to the NMDA receptor (51). Dlg3 is recognized as the first cognitive disability gene associated with glutamate receptor signaling and transportation (52). The expression of SAP102 is decreased in hippocampus of APP/PS1 mice and is in decline in the inferior temporal cortex and occipital cortex of patients with AD (53, 54). In this study, we showed that Smad4 inhibition increased the expression of Dlg3 by directly binding to the –1,049 to –776 bp of the Dlg3 promoter, and whether the protective effects of Smad4 inhibition are mediated by Dlg3 induction will be investigated.

Aβ and p-tau in serum and CSF are considered the most promising biomarkers for AD diagnosis and prognosis (55–57). Currently, the main diagnostic methods for AD are based on PET imaging and CSF analysis, which are relatively expensive and highly invasive; therefore, there is an urgent need to explore new diagnostic methods that can help diagnose AD at an early stage with high specificity at a lower cost (58, 59). Emerging evidence has shown that miRNAs are alternative targets for AD diagnosis partly due to relatively higher stability and sensitivity. A set of serum miRNAs is downregulated in patients with AD compared with those of the control group (60, 61). For example, miR-455-3p is significantly increased in the serum and brains of patients with AD and plays critical roles in the AD pathogenesis (62). Serum miR-501-3p is downregulated in patients with AD and closely correlated with neuropsychological scores (63). A novel 9-miRNA signature, including hsa-miR-22-3p, can be used as a biomarker for AD diagnosis by next-generation sequencing (64). MiR-431 has been shown to regulate the apoptosis of cardiomyocytes and chondrocytes and suppress proliferation and metastasis of cancers (65–69). In our study, we found that plasma miR-431 was reduced in 6-, 9-, and 12-month-old APP/PS1 mice and decreased in patients with aMCI and AD. To the best of our knowledge, this is the first report to reveal that plasma miR-431 might be an alternative biomarker for AD diagnosis. Overall, our data have indicated that miR-431/Smad4 plays an important role in the pathogenesis of AD, and upregulating miR-431 and/or inhibiting Smad4 might be a potential therapeutic strategy for AD treatment.

## Methods

### Participants.

The participants including 23 healthy controls (HCs), 20 patients with aMCI, and 25 patients with AD were recruited from the Department of Neurology in Nanjing Drum Tower Hospital. All participants underwent clinical information collection, a neurological examination, a brain MRI scan, and neuropsychological tests including the Mini-Mental State Examination (MMSE) and Montreal Cognitive Assessment. All study participants were recruited based on the Alzheimer’s Disease Neuroimaging Initiative (ADNI) inclusion and exclusion criteria (70, 71). In the ADNI, HCs were required to have a Clinical Dementia Rating (CDR) score of 0 and MMSE scores ≥ 26 (inclusive) without memory complaints. Inclusion and exclusion criteria for ADNI aMCI included (i) subjective memory impairment corroborated by participant and an informant; (ii) objective memory performances documented by an Auditory Verbal Learning Test delayed recall score ≤ 1.5 SD of age- and education-adjusted norms (cutoff of ≤4 correct responses on 12 items for ≥8 years of education); (iii) MMSE scores ≥ 24; (iv) CDR score of 0.5; (v) no or minimal impairment in activities of daily life; and (vi) absence of dementia, or not sufficient to meet the Neurological Disorders and Stroke–Alzheimer Disease and Related Disorders (NINCDS–ADRDA) Alzheimer’s Criteria. Inclusion and exclusion criteria for AD included memory complaints in daily life, CDR scores between 0.5 and 2.0, MMSE scores ≤ 26, and the criteria for probable AD diagnosis according to NINCDS–ADRDA. In addition, participants with a significant psychiatric illness and neurologic condition history were excluded (e.g., depression, epilepsy, stroke, Parkinson’s disease, traumatic brain injury).

Demographic and neuropsychological data of the participants are shown in Supplemental Table 1. The study was approved by the Nanjing Drum Tower Hospital Ethics Committee, complied with the Helsinki Declaration II, and included written informed consent from all participants.

### Animals and treatment.

Six-month-old male APP/PS1 and age-matched WT littermates were provided by the Model Animal Research Center of Nanjing University. Mice were housed in polypropylene cages on a 12-hour light/12-hour dark cycle, with access to water and food ad libitum. MiR-431 overexpression sequence was synthesized and inserted into lentiviral vector GV309 (hU6-MCS-Ubiquitin-EGFP-IRES-puromycin) (Lv-miR-431) and the scramble control (Lv-con) from Shanghai Genchem. Smad4 overexpression lentivirus (CMV-Smad4-3flag-EF1a-hScarlet-T2A-Puromycin) (Lv-Smad4) and the scramble control (CMV-scramble-3flag-EF1a-hScarlet-T2A-Puromycin) (Lv-con2) were acquired from Shanghai Genchem. The AAV knocking down Smad4, rAAV-hSyn-EGFP-5’miR-30a-sh (Smad4)-3’-miR30a-WPREs, AAV2/9 (AAV-sh-Smad4), and the corresponding control, rAAV-hSyn-EGFP-5’ miR-30a-sh (scramble)-3’-miR30a-WPREs, AAV2/9 (AAV-con), were packaged by BrainVTA. Lv-miR-431 (1 × 10^9^ transducing units/mL, 200 nL/min, 2 μL), Lv-Smad4 (1 × 10^9^ transducing units/mL, 200 nL/min, 2 μL) or AAV-sh-Smad4 (5 × 10^12^ viral genomes/mL, 20 nL/min, 200 nL) was slowly injected into the bilateral hippocampus CA1 (anterior-posterior –1.85 mm, medial-lateral ±1.5 mm, dorsal-ventral +1.65 mm from bregma) of 6-month-old APP/PS1 mice using a stereotaxic apparatus as previously described (10, 72). Behavior tests were performed to evaluate memory functions 1 month following the treatment. All animal experiments were approved by the Animal Care Committee of Nanjing University.

### Cell culture and treatment.

Primary cortical neurons were isolated from E15–E17 embryos of WT mice, as previously described (73), and seeded on poly-d-lysine–coated plates. Cells were cultured in neurobasal medium with B27 (Invitrogen) and 200 mM glutamine at 37°C in a humidified 5% CO_2_ incubator and treated with Lv-miR-431 (MOI = 20) at day in vitro (DIV) 4. Primary hippocampal neurons were extracted from early postnatal (P0–P1) WT and APP/PS1 mouse hippocampus (74). The cells were plated onto poly-d-lysine–coated plastic coverslips in 24-well plates at 50,000 cells per well for synaptic density quantification or poly-d-lysine–coated 6-well plates at 20,000 cells for protein extraction. A total of 5 μM cytosine arabinoside was used to inhibit the proliferation of non-neuronal cells at DIV 1. Lv-con was added into the culture medium of WT hippocampal neurons or APP/PS1 hippocampal neurons at DIV 4, and these 2 groups were named Lv-WT and Lv-con, respectively. Lv-miR-431 was added into the culture medium of APP/PS1 hippocampal neurons at DIV 4, and this group was named Lv-miR-431. The culture medium was fully replaced 16 hours after the lentivirus transfection. At DIV 15, the levels of PSD-95 and SYN1 were determined by immunofluorescence staining and Western blot. HEK293T cells, which were maintained in our lab (75), were grown in DMEM (Invitrogen) supplemented with 10% fetal bovine serum (Hyclone) at 37°C with 5% CO_2_.

### Behavior tests.

The behavior tests were performed as previously described (10), and all the behavior tests were performed in a double-blind manner.

### OF tests.

OF tests were used to assess locomotor activity and anxiety of the mice. The open field area (consisting of a 48 cm × 48 cm × 36 cm box) included a central square of 24 cm × 24 cm named the central area and 4 corner squares of 12 cm × 12 cm named corner area. The total distance traveled in the open field and the time spent in the each square were measured and recorded by ANY-maze software (Stoelting) in a 600-second session.

### NOR test.

Mice were allowed to walk freely in a 30 cm × 30 cm × 45 cm white box without objects within 5 minutes twice a day for 3 days before the NOR tests. In the training trial, mice were placed in the box containing 2 of the same objects (object A1 and object A2) and allowed to explore freely for 10 minutes. In the test trial, object A2 was replaced by a novel object (object B). Then, mice were allowed to explore the 2 objects (object A1 and object B) for another 5 minutes. The time exploring the objects was recorded by a visual tracking system and respectively named as EA and EB. The discrimination index was calculated as EB/(EA+EB).

### MWM tests.

The MWM tests were used to access spatial memory functions. In brief, the test was divided into 2 stages for a total of 6 days. During the acquisition trial (day 1 through day 5), mice were trained to find a platform under the water within 60 seconds, and the latency was recorded by using ANY-maze software. Mice were trained once a day in each of the 4 quadrants. In the probe trial, mice were allowed to swim in the maze without a platform for 1 minutes. Then the number of platform crossings, the latency to find the target quadrant, and the time spent in target quadrant were recorded.

### Contextual FC tests.

Contextual FC tests were performed using a conditioning chamber (Panlab). In the training trial, mice were allowed to move freely in the chamber for 3 minutes and then given a shock (2 seconds, 0.75 mA), and the mice were then allowed to stay in the chamber for another 2 minutes. Twenty-four hours after training, the mice were subjected to testing of the hippocampus-dependent memory. Mice were put into the same chamber as the previous day for 5 minutes, and the freezing time was recorded by a tracking system (Panlab).

### Quantitative real-time PCR.

MiRNeasy Serum/Plasma Kit (QIAGEN) was used to extract plasma miRNAs, and RNeasy Mini Kit (QIAGEN) was used to extract total tissue RNA. For quantitative real-time PCR (qRT-PCR) of miRNAs, a reaction mix of 10 μL TaqMan universal master mix, with UNG (2×), 8 μL RNase-free water, 1 μL specific TaqMan Advanced miRNA Assays (20×) (Thermo Fisher Scientific, catalog 001979), and 1 μL cDNA was added to a 96-well PCR plate, and PCR amplifications were performed on a Step One Plus PCR system (Applied Biosystems). Cel-miR-39 (Thermo Fisher Scientific, catalog 000200) and U6 snRNA (Thermo Fisher Scientific, catalog 001973) were used as internal references for plasma and tissue, respectively. The relative mRNA of Smad4 and Dlg3 was detected by qRT-PCR using SYBR Green Premix Pro Taq HS qPCR Kit (AG11701, ACCURATE BIOTECHNOLOGY, HUNAN, Co.,Ltd). The primers were as follows: MiR-431 forward: AGGTGTCTTGCAGGCCGT, reverse: GTGCGTGTCGTGGAGTCG; U6 forward: GCTTCGGCAGCACATATACTAAAAT, reverse: CGCTTCACGAATTTGCGTGTCAT; Smad4 forward: ACACCAACAAGTAACGATGCC, reverse: GCAAAGGTTTCACTTTCCCCA; Dlg3 forward: ACATTCTGCACGTCATTAACGC, reverse: ATGTCACTCCCTTCAGGTTCT; GAPDH forward: GCCAAGGCTGTGGGCAAGGT, reverse: TCTCCAGGCGGCACGTCAGA.

### Electrophysiology.

Mice were sacrificed under isoflurane anesthesia, and the fresh hippocampal slices (300 μm) were prepared as previously described (10). Then the slices were transferred into the microelectrode array, continuously perfused with oxygenated ACSF (2 mL/min), and maintained at 32°C for recording. Field excitatory postsynaptic potentials (fEPSPs) in the CA1 stratum radiatum were recorded by using MEA-2100-60-System (Multi Channel Systems). I/O curves were obtained with incremental stimulation intensity from 10 to 100 μA. To evaluate the input/output relationships of synapses, the amplitudes of fEPSPs were plotted as a function of fiber volley amplitudes. In the LTP experiments, the stimulation intensity was the half of the maximum evoked response. The LTP was induced by high-frequency stimulus (three 100 Hz trains, 1 second duration, 10 second interval time). Initial fEPSP slopes were normalized in each experiment using the averaged slope value during the control period. LTP-Director software was used for acquiring data, and LTP-Analyzer software was used for data analysis.

### Golgi staining.

FD Rapid GolgiStain Kit (FD Neurotechnologies) was used for Golgi staining as previously described (10, 76). In brief, brain tissues of mice were immersed in 8 mL mixed solution including 4 mL solution A and 4 mL solution B for 2 weeks followed by solution C for at least 3 days at room temperature in the dark. Then the tissues were cut into 100 μm sections using a Leica CM1950 cryostat. The brain sections were flushed in a mixed solution including solution D and E. Then the slices were rinsed, dehydrated, dried, cleared, and covered with coverslips. Olympus BX51 microscope was used for acquiring the images. The pyramidal neurons in the CA1 region of the hippocampus were analyzed. At least 3 dendrites were randomly selected, and 3 segments (at least 30 μm) were randomly chosen per neuron from the dendrites for each mouse. And the numbers of spines per 10 μm were counted in a blinded manner by ImageJ software (NIH). The specific steps follow: 1) Open the images in ImageJ, set scale bar, and select Plugins, Analyze and Cell Counter; 2) click Initialize and select Type 1, and start counting with the right mouse button; 3) count the number of spines, and enlarge the target area; 4) click Straight with the right mouse button, and select Segmented Line, 5) draw lines on the counted dendrites, and 6) click Analyze and Measure. We counted 3 dendrites from per mice and calculated the average.

### Electron microscopy.

Mice were sacrificed under isoflurane anesthesia, and the hippocampus was isolated and cut into 1 mm^3^ pieces. Then the pieces were fixed with 0.1 M sodium cacodylate buffer (pH 7.4) containing 2.5% glutaraldehyde for 2 hours at room temperature, rinsed with 0.1 M PBS (pH 7.4), and postfixed in 1% osmium tetroxide in the dark for 2 hours. Subsequently, the pieces were dehydrated in graded increasing ethanol and embedded in epoxy resin. The embedding models with resin and samples were moved into a 65°C oven to polymerize for more than 48 hours; then the resin blocks were cut to 60–80 nm thin on the ultramicrotome (Leica) and stained with 2% uranyl acetate and 2.6% lead citrate. The images were captured by a Hitachi 7100 electron microscope. The number of synapses in hippocampus CA1 was analyzed by an experimenter following a protocol blinded to treatment and genotype using ImageJ software (77, 78).

### Luciferase activity assay.

Smad4 3′-UTR and mutant Smad4 3′-UTR were inserted into the pGL3-CMV-LUC-MCS vector (Genomeditech). In the luciferase reporter assay, HEK293T cells were infected by Lv-miR-431 for 72 hours, then transfected with Smad4 3′-UTR or mutant Smad4 3′-UTR for another 24 hours using Lipofectamine 3000 (Invitrogen, Thermo Fisher Scientific). Dlg3 promoter (–2,500 to ~+2 bp) and its truncated form (–1,049 to –776 bp deleted) were inserted into the pGL3-promoter vector with a luciferase reporter gene (Genomeditech). Smad4 overexpression plasmid and control plasmid were purchased from Genomeditech. HEK293T cells were transfected with Smad4 overexpression plasmid, Dlg3 promoter plasmid, or truncated plasmid for 24 hours by using Lipofectamine 3000. PhRL-CMV Renilla was also transfected into HEK293T cells as an internal reference. The luciferase activity was determined using the Promega Bright-N-Glo system.

### Western blot.

The protein of hippocampus and primary neurons was extracted and quantified as previously described (72). Approximately 40 μg proteins were separated by 10% SDS-PAGE and then transferred to PVDF membranes. The membranes were incubated with the following primary antibodies: rabbit anti-Smad4 (1:1,000, Cell Signaling Technology, catalog 46535), rabbit anti-SAP102 (1:1,000, Cell Signaling Technology, catalog 47421), mouse anti-NMDAR1 (1:1,000, Abcam, catalog ab134308), rabbit anti-NMDAR2A (1:1,000, Abcam, catalog ab169873), rabbit anti-NMDAR2B (1:1,000, Abcam, catalog ab65783), rabbit anti-GluA1 (1:1,000, Abcam, catalog ab31232), rabbit anti-GluA2 (1:1,000, Abcam, catalog ab133477), rabbit anti-synapsin1 (1:1,000, Abcam, catalog ab64581), rabbit anti-Homer1 (1:1,000, Abcam, catalog ab184955), rabbit anti-CaMKII (1:1,000, Abcam, catalog ab52476), rabbit anti–PSD-95 (1:1,000, Abcam, catalog ab18258), rabbit anti-synaptophysin (1:20,000, Abcam, catalog ab32127), and rabbit anti-GAPDH (1:5,000, Bioworld, catalog AP0063) overnight at 4°C, then incubated with HRP-conjugated secondary antibodies for 1 hour at room temperature: goat anti-rabbit IgG (H+L) HRP (1:5,000, Bioworld, catalog BS13278), goat anti-mouse IgG (H+L) HRP (1:5,000, Bioworld, catalog BS12478). The protein bands were visualized with the ECL Detection Kit (MilliporeSigma). Images were acquired using the Gel-Pro system (Tanon Technologies), and ImageJ software was used to analyze the intensity of each band. The specific steps follow: 1) open Western blot images in ImageJ, select Rectangular Selections tool from the ImageJ toolbar, and select first Western band; 2) press Ctrl + 1 and Ctrl + 3 to open histogram; 3) choose the Straight Line selection tool from the ImageJ toolbar, and draw a line on the bottom of pictures to make them completely enclosed; 4) select the Wand tool from the ImageJ toolbar, and click inside the histogram once, one by one (the results will show up in the Results block diagram); 5) copy the numerical values of target genes into Excel and divide by their respective numerical values of GAPDH.

### Immunofluorescence staining.

Immunofluorescence assay was used to detect the Aβ load in the hippocampus of APP/PS1 mice and measure the synaptic density quantification in the primary hippocampal neurons. For brain sections, the mice were anesthetized, then perfused with 0.9% saline and 4% paraformaldehyde, and then the brains were removed and subjected to gradient dehydration, followed by cutting into 20 μm sections with a Leica CM1950 cryostat. The brain slices were treated with 0.25% PBS– Triton X-100 (PBS-T) for 15 minutes, washed 3 times with PBS, blocked with 2% BSA for 2 hours, and incubated with primary antibody mouse anti-beta amyloid 6e10 (1:200, BioLegend, catalog 803001) and primary antibody mouse anti-beta amyloid 82e1 (2 μg/mL, Immuno-Biological Laboratories, catalog 10323) at 4°C overnight. Then, the slices were incubated with appropriate fluorescent secondary antibody for 2 hours at room temperature. DAPI was used to stain the nuclei. An inverted fluorescence microscope (Olympus IX73) was used to capture the images. The areas of Aβ plaque in the images were analyzed by ImageJ software. The specific steps follow: 1) open the staining image in ImageJ, select Image tools from the ImageJ toolbar, and click type and 8-bit; 2) select Image, Adjust and Threshold, and adjust the upper limit value and lower limit value in the threshold to make red spots cover the Aβ plaque accurately.

For culture of cells on the coverslips, the cells were washed with PBS 3 times and fixed with 4% paraformaldehyde for 10 minutes, followed by treating with 0.25% PBS-T for 15 minutes, washing 3 times with PBS, and then blocking with 2% BSA for 2 hours and incubating with primary antibody mouse anti-Synapsin1 (1:500, SYSY, catalog 106011) or primary antibody rabbit anti–PSD-95 (1:500, Abcam, catalog 18258) at 4°C overnight. Then, the slices were incubated with appropriate fluorescent secondary antibody for 2 hours at room temperature. The images were captured by a confocal fluorescence microscope (Olympus FV3000). Synaptic density quantification was analyzed by using ImageJ software. The specific steps follow: 1) open the single dendrite image in ImageJ, click Process, and select Find Maxima; 2) set definite values of noise tolerance, e.g., 15, and tick Preview Point Selection, and make the cross marks cover fluorescence signal accurately to ensure synaptic density values will show up. The corresponding synaptic density values were converted to the synaptic density value at 100 μm length.

### Aβ ELISA.

Soluble and insoluble ingredients of Aβ were detected as previously reported (10, 72). The frozen tissues were homogenized in 15 volumes (*w/v*) of TBS homogenization buffer, which contained phosphatase and protease inhibitor cocktails (Thermo Fisher Scientific), and centrifuged at 100,000*g* for 1 hour at 4°C. The supernatant fraction was collected as TBS-soluble fraction, and the sediment fraction was resuspended in 15 volumes (*w/v*) of 1% Triton X-100/TBS (TBS-X). And then the samples were incubated on ice for 30 minutes followed by centrifuging at 100,000*g* for 1 hour at 4°C. The supernatant fraction was collected as TBS-X–soluble fraction, and the sediment fraction was resuspended with 15 volumes (*w/v*) of 70% FA. After the samples were centrifuged at 100,000*g* for 1 hour at 4°C, the supernatant fraction was neutralized by 20 volumes 1 M Tris base (pH 11), which was named FA-soluble fraction. The protein concentration of TBS-soluble fraction and TBS-X–soluble fraction was measured by BCA protein assay kit (Thermo Fisher Scientific). And protein concentration of FA-soluble fraction was measured by a Bradford protein assay kit (Beyotime). The Aβ_40_ and Aβ_42_ levels were quantified with Quantikine ELISA Human Amyloid β aa1-40/aa1-42 immunoassay kits (R&D Systems) according to the manufacturer’s protocol.

### Cut&Tag assay.

Cut&Tag assay was performed by using Hyperactive Universal CUT&Tag Assay Kit for Illumina (TD903, Vazyme Biotech Co.,Ltd) as described previously (79). Briefly, native nuclei were purified from hippocampus tissue as previously mentioned (80). Concanavalin A–coated magnetic beads (Vazyme Biotech Co.,Ltd) were added and incubated at room temperature for 10 minutes. Bead-bound cells were incubated with rabbit anti-Smad4 (1:50, Cell Signaling Technology, catalog 46535) or IgG control antibody (1:50, MilliporeSigma, catalog 12-370) on a rotating platform overnight at 4°C. Secondary antibody (anti-rabbit IgG antibody, MilliporeSigma) was added, and pA-Tn5 adapter complex was prepared in dig-med buffer. Then cells were resuspended in tagmentation buffer (10 mM MgCl_2_ in dig-med Buffer) and incubated at 37°C for 1 hour. DNA was purified using phenol-chloroform-isoamyl alcohol extraction and ethanol precipitation. The size distribution of libraries was determined by Agilent 4200 TapeStation analysis, and sequencing was performed in the Illumina NovaSeq 6000 using 150 bp paired-end reads following the manufacturer’s instructions. The bam file was generated by the unique mapped reads as an input file, using macs2 software for call peak with cutoff value < 0.05.

### ChIP.

ChIP was performed according to the instructions of SimpleChIP Plus Enzymatic Chromatin IP Kit (Cell Signaling Technology). Briefly, the fresh 50 mg hippocampal tissue was cross-linked in 1.5% formaldehyde for 20 minutes. Dounce homogenizer was used to ground into a single-cell suspension, and then the chromatin was digested to 150–900 bp by using micrococcal nuclease. After sonicating 3 times for 20 seconds, the samples were incubated with 1 μg of rabbit anti-Smad4 (Cell Signaling Technology, catalog 46535) antibody or control rabbit IgG (Cell Signaling Technology, catalog 2729) on a rotary table overnight at 4°C. Then 30 μL of ChIP-Grade Protein G Magnetic Beads were added and incubated for 2 hours. DNA was eluted from antibody/Protein G Magnetic Beads, and the cross-links were unlocked and purified using spin columns for qPCR.

Sequences of primers for ChIP–real-time PCR were forward: TAGTGGGTAGAGCAGGGAG; reverse: GTGTCCAGAGATGTTCCACT.

### Statistics.

All data were expressed as the mean ± SEM of at least 3 independent experiments and analyzed by SPSS 20.0. Shapiro-Wilk test was used to test the normality assumption of the data. Two-tailed unpaired Student’s *t* test was used to compare differences between 2 groups if the data were normal distributed, while Mann-Whitney *U* test was applied to compare the non-normally distributed variables. For more than 2 groups, statistical difference was analyzed by 1-way or 2-way ANOVA followed by Bonferroni’s post hoc test or by the Kruskal-Wallis test followed by Dunn’s multiple-comparison test. Pearson’s correlation analysis was used to analyze the correlation of Smad4 with miR-431. *P* < 0.05 was considered statistically significant.

### Study approval.

All mouse experiments were approved by the Animal Care and Ethics Committee of the Model Animal Research Center of Nanjing University. The study was approved by the Nanjing Drum Tower Hospital Ethics Committee, complied with the Helsinki Declaration II, and included written informed consent from all participants.

### Data availability.

The Cut&Tag data are deposited in the NCBI Gene Expression Omnibus database with the accession number GSE231985.

## Author contributions

XZ designed research; JG, ZX, SS, L Yu, WT, RQ, PL, XD, ZL, XB, L Ye, YX, and XZ performed experiments; JG, ZX, and SS analyzed data; JG and XZ wrote the manuscript; and PL, ZL, and YX provided valuable comments and revised the manuscript. All authors read and approved the final version of manuscript.

## Supplementary Material

Supplemental data

## Version 1 05/16/2023

In-Press Preview

## Version 2 06/22/2023

Electronic publication

## Version 3 06/28/2023

The affiliation addresses were updated
