# Supplementary material for: MiR-431 attenuates synaptic plasticity and memory deficits in APPswe/PS1dE9 mice
Source: JCI Insight. 2023 Jun 22;8(12):e166270. doi: 10.1172/jci.insight.166270 (PMC10371242; doi:10.1172/jci.insight.166270)
Supplement: Supplemental data [file jciinsight-8-166270-s240.pdf]

Supplementary data

MiR-431 attenuates synaptic plasticity and memory deficits in APPswe/PS1dE9 mice

Jianwei Ge[1,2,3,4#], Zhiwei Xue[1,2,3,4#], Shu Shu[1,2,3,4#], Linjie Yu[1,2,3,4], Ruomeng Qin[1,2,3,4], Wenyuan Tao[1,2,3,4], Pinyi Liu[1,2,3,4], Xiaohong Dong[1], Zhen Lan[5], Xinyu Bao[1,2,3,4], Lei Ye[1,2,3,4], Yun Xu[1,2,3,4,5], Xiaolei Zhu[1,2,3,4,5*]

[1]Department of Neurology, Drum Tower Hospital, Medical School and The State Key Laboratory of Pharmaceutical Biotechnology, Nanjing University, Nanjing, Jiangsu, 210008, PR China

[2]Institute of Brain Sciences, Institute of Brain Disorder Translational Medicine, Nanjing University, Nanjing, Jiangsu 210008, China

[3]Jiangsu Key Laboratory for Molecular Medicine, Medical School of Nanjing University, Nanjing, Jiangsu 210008, PR China

[4]Nanjing Neuropsychiatry Clinic Medical Center, Nanjing, Jiangsu 210008, PR China

[5]Department of Neurology, Drum Tower Hospital of Nanjing Medical University, Nanjing, Jiangsu 211166, PR China

[#] Jianwei Ge, Zhiwei Xue and Shu Shu contributed equally to this study.

∗ Correspondence to: Xiaolei Zhu, M.D., Ph.D., Department of Neurology, Drum Tower Hospital, Medical School of Nanjing University, 321 Zhongshan Road, Nanjing, Jiangsu 210008, P.R. China. Tel.: +86 25 68182212; Fax: +86 25 83105208;

E-mail address: zhuquelee@126.com.

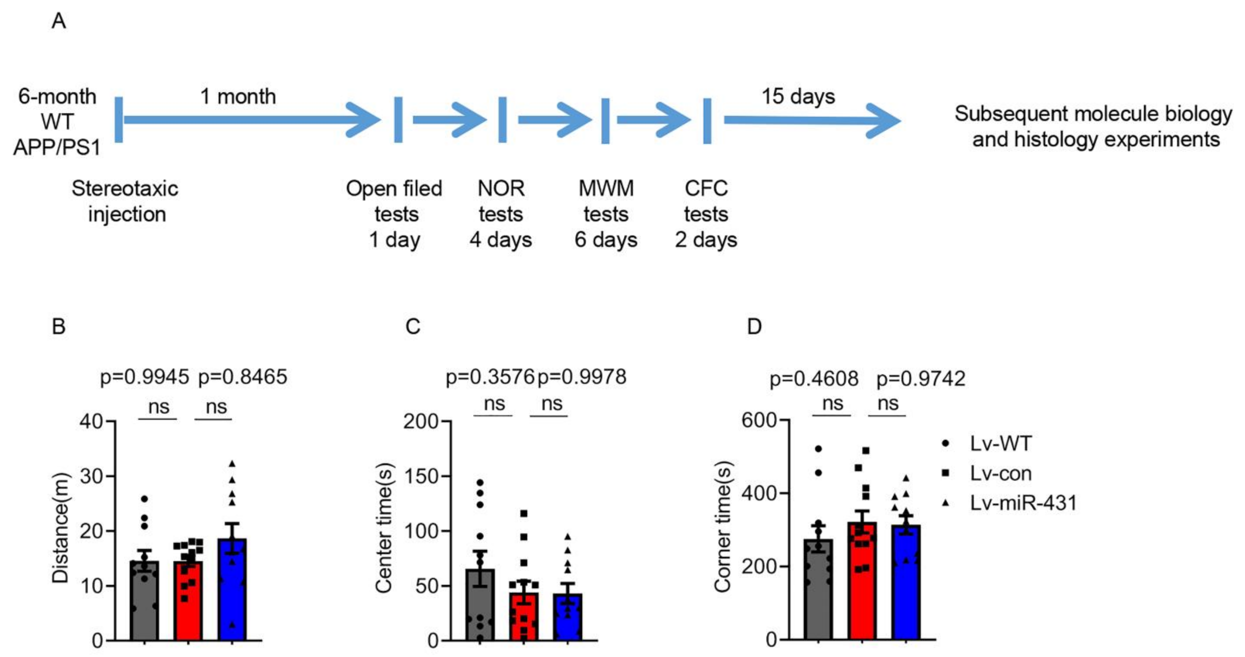

Figure S1 MiR-431 overexpression doesn't modulate the motor function and emotional state of 6-month-old APP/PS1 mice. (A) The flow chart of the experiments. The total distance (B), time spent in the center (C) and corner area (D) were recorded of Lv-miR-431 treated APP/PS1 mice by Any-maze software in the OF test. n = 11-12 for each group, ns: not significant. All data were presented as means ± SEM. One-way ANOVA (B-D) was used.

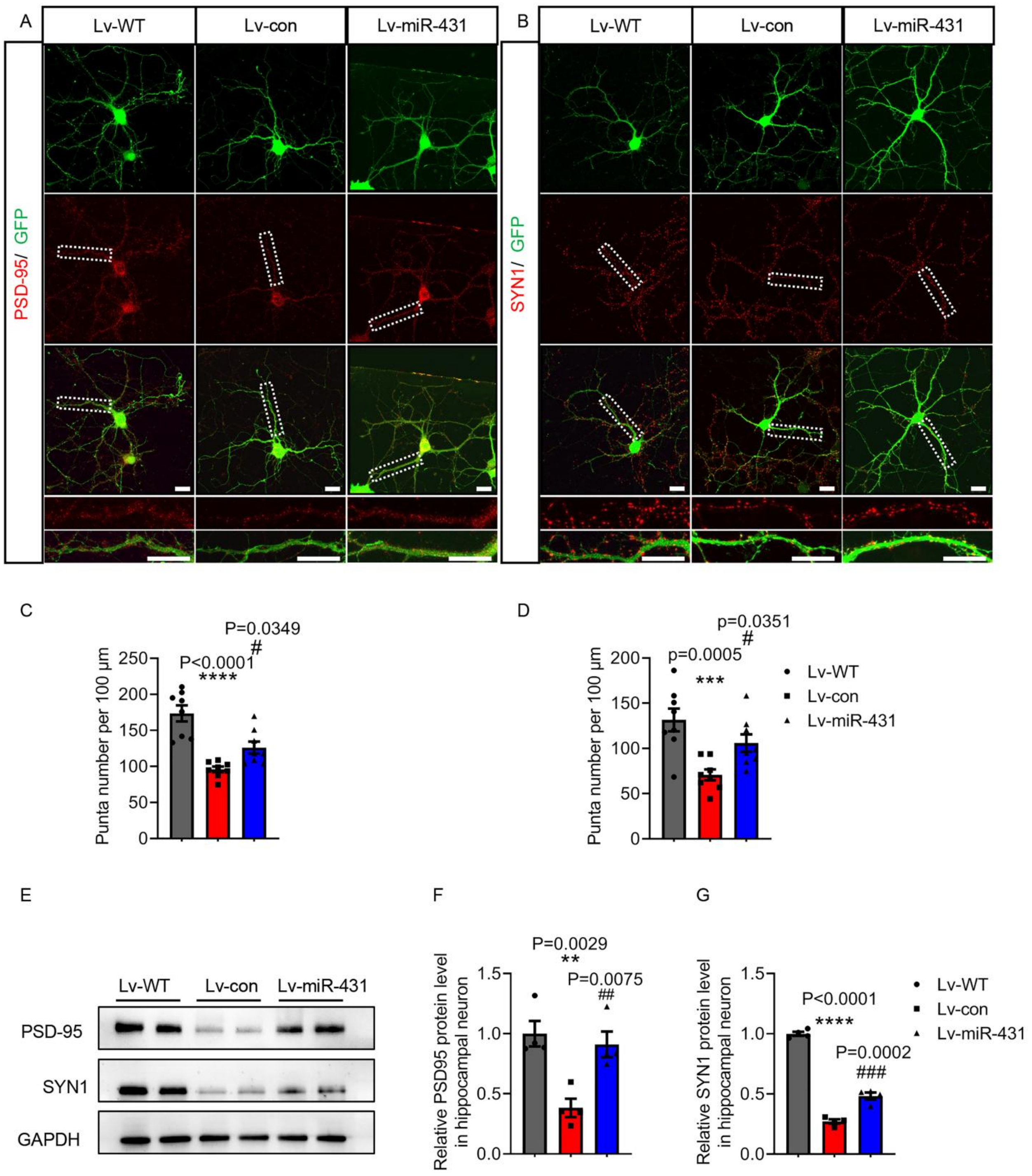

Figure S2 MiR-431 overexpression improves the synaptic density, the PSD-95 and SYN1 protein levels in APP/PS1 hippocampal neurons. (A) Representative confocal images of PSD-95 (red) and GFP (green). Upper bar = 20μm, lower bar = 20μm. (B) Representative confocal images of SYN1 (red) and GFP (green). Upper bar = 20μm, lower bar = 20μm. (C) Quantification of PSD-95-positive puncta per 100μm of GFP-positive neurites. n = 8 neurons per group. (D) Quantification of SYN1-positive puncta per 100μm of GFP-positive neurites. n = 8 neurons per group. (E) Representative western blot of PSD-95 and SYN1 in WT or APP/PS1 hippocampal neurons. (F) Quantification of PSD-95 blots. n = 4. (G) Quantification of SYN1 blots. n = 4. $^{**}p<0.01$, $^{***}p<0.001$, $^{****}p<0.0001$ vs. Lv-WT group; # $p< 0.05$, ## $p< 0.01$, ### $p<0.001$ vs. Lv-con group. All data were presented as means ± SEM. One-way ANOVA (C, D, F and G) was used.

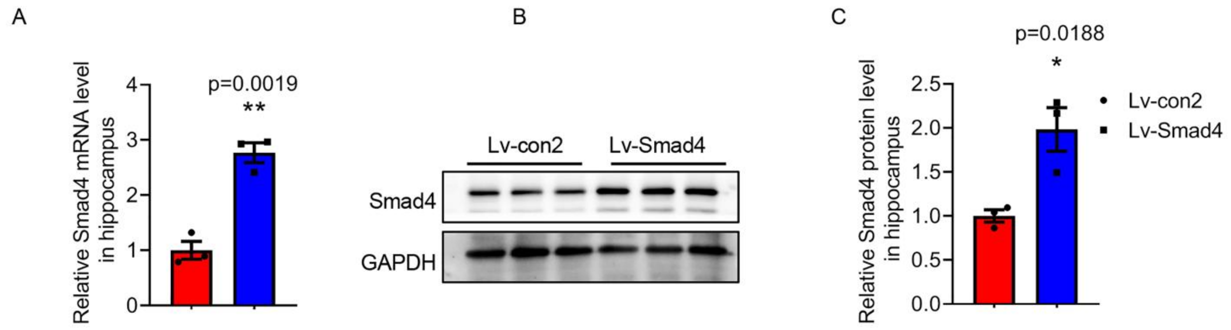

Figure S3 Smad4 overexpression efficiency in Lv-Smad4 treated hippocampus of APP/PS1 mice. (A) The mRNA level of Smad4 in Lv-Smad4 treated hippocampus. n = 3 (B, C). The protein level of Smad4 in Lv-Smad4 treated hippocampus. n = 3, * p<0.05, ** p<0.01 vs. Lv-con2. All data were presented as means ± SE

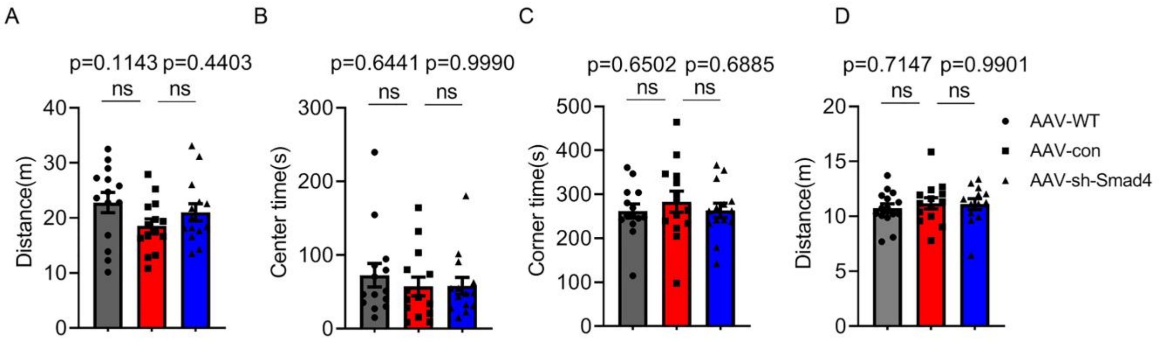

Figure S4 Smad4 inhibition doesn't affect the motor function and emotional state of 6-month-old APP/PS1 mice. The total distance (A), time spent in the center (B) and corner area (C) were recorded of AAV-sh-Smad4 treated APP/PS1 mice by Any-maze software in the OF test. (D) Distance in MWM tests was detected in the acquisition trial, n = 14, ns: not significant. All data were presented as means ± SEM. One-way ANOVA (A-D) was used.

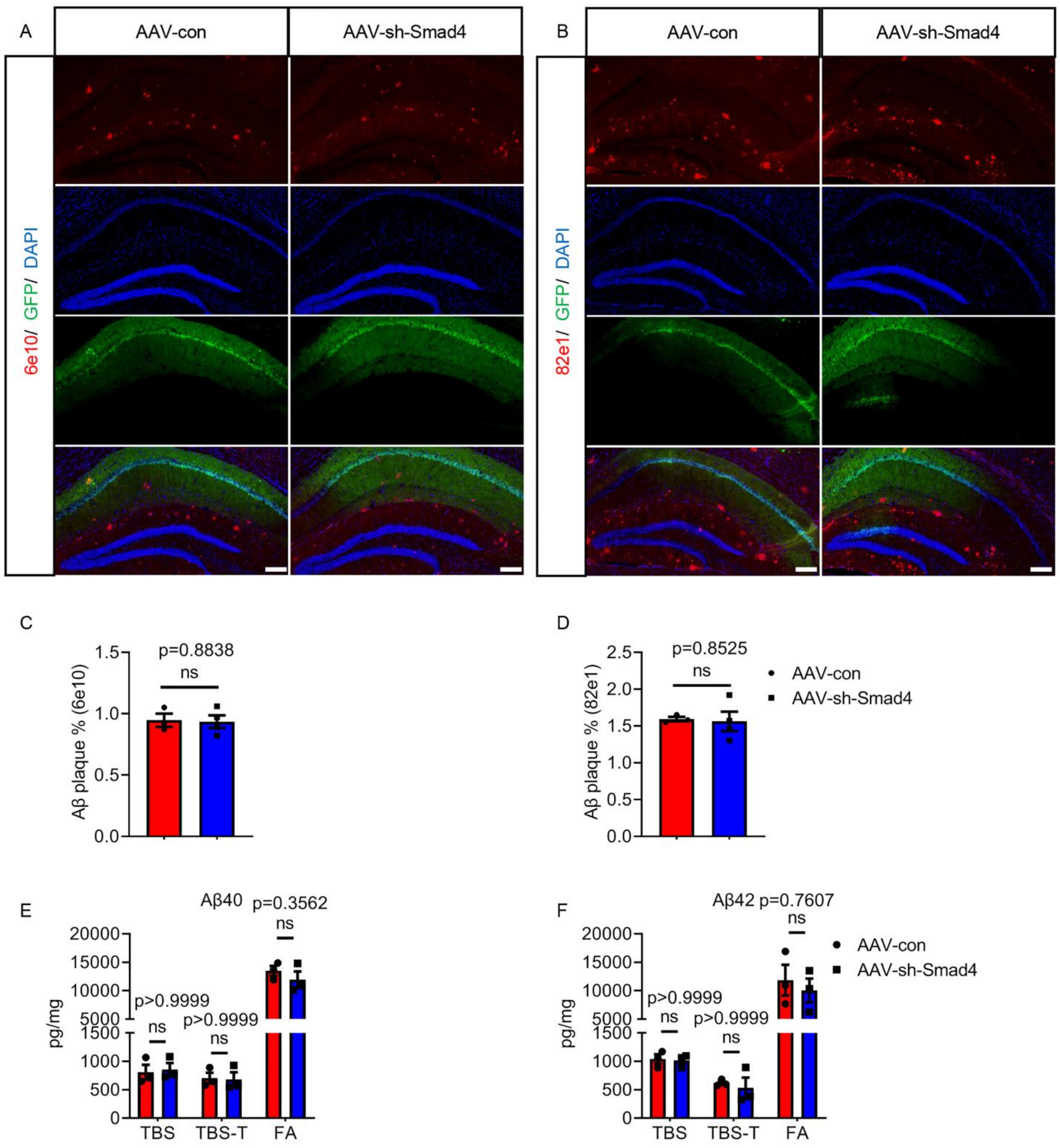

Figure S5 Smad4 inhibition doesn't affect the Aβ levels in the hippocampus of 6-month-old APP/PS1 mice. (A) The level of Aβ 6e10 in the hippocampus of AAV-sh-Smad4 treated APP/PS1 mice were detected by immunofluorescence staining. (B) The level of Aβ 82e1 in the hippocampus of AAV-sh-Smad4 treated APP/PS1 mice were detected by immunofluorescence staining. (C) Quantitative analysis of the percentage of Aβ 6e10 positive area. n = 3-4 mice per group. Bar = 200μm. (D) Quantitative analysis of the percentage of Aβ 82e1 positive area. n=3-4 mice per group. Bar = 20 μm. The protein levels of TBS-soluble, TBS-T-soluble and FA-soluble $A\beta_{40}$ (E) and $A\beta_{42}$ (F) were measured by ELISA in the hippocampus of AAV-sh-Smad4 treated APP/PS1 mice. n=3, ns: not significant. All data were presented as means ± SEM. Two-tailed unpaired Student's t test (C, D) and two-way ANOVA (E, F) were used.

Supplement Table 1. Demographic and neuropsychological data.

Items	HC (n=23)	aMCI (n=20)	AD (n=25)	*p*	*p*(Mann-Whiney U Test)		
					NC versus aMCI	NC versus AD	aMCI versus AD
Demographics							
Age(y)	63.33±1.5	68.65±2.4	66.28±1.5	0.1383	0.1200	0.4434	0.6259
Education(y)	11.78±0.9	10.88±1.0	6.520±0.85	0.0011*	0.4304	0.0093*	0.0003*
Gender (male/female)	12/11	10/10	10/15	0.7747	>0.9999	0.5572	0.7715
General cognition							
MMSE	29.17±0.2	26.93±0.7	14.00±1.6	<0.0001*	0.0078*	<0.0001*	<0.0001*
MoCA	24.39±0.7	20.00±1.1	8.83±1.5	<0.0001*	0.0019*	<0.0001*	<0.0001*

Abbreviations: HC, Healthy control; aMCI, amnestic Mild cognitive impairment; AD, Alzheimer's disease; MMSE, Mini-Mental State Exam; MoCA, Montreal Cognitive Assessment.

Values were presented as the average ± standard error (SE).

p-value, the data do not satisfy the normal distribution in the one-way ANOVA, so the p-value is obtained by Kruskal-Wallis test. *p* (Mann-Whiney U Test) was used here due to the fact that the data were not normally distributed in the Post-hoc tests. * indicates a statistical difference between groups, $p < 0.05$.
